# Portable gas chromatography–mass spectrometry method for the in-field screening of organic pollutants in soil and water at pollution incidents

**DOI:** 10.1007/s11356-023-28648-w

**Published:** 2023-07-27

**Authors:** Denise Duff, Chris Lennard, Yarong Li, Christopher Doyle, Katelyn J. Edge, Ian Holland, Kevin Lothridge, Paul Johnstone, Paul Beylerian, Val Spikmans

**Affiliations:** 1 School of Science, Western Sydney University, Locked Bag 1797, Penrith, NSW 2751 Australia; 2 Department of Planning and Environment, Environment Protection Science Branch, Building 1, 480 Weeroona Road, Lidcombe, NSW 2141 Australia; 3 New South Wales Environment Protection Authority, Incident Management and Environmental Health Branch, Locked Bag 5022, Parramatta, NSW 2124 Australia; 4 Global Forensic and Justice Center @ Florida International University, 8285 Bryan Dairy Road. Suite 125, Largo, FL 33777 USA; 5 Operations Capability Directorate, Fire & Rescue NSW, 1 Amarina avenue, Greenacre, NSW 2190 Australia

**Keywords:** Environmental regulation, Person portable equipment, Mobile laboratory, Risk assessment, Risk management, Environmental forensics, Field analysis

## Abstract

Environmental pollution incidents generate an emergency response from regulatory agencies to ensure that the impact on the environment is minimised. Knowing what pollutants are present provides important intelligence to assist in determining how to respond to the incident. However, responders are limited in their in-field capabilities to identify the pollutants present. This research has developed an in-field, qualitative analytical approach to detect and identify organic pollutants that are commonly detected by regulatory environmental laboratories. A rapid, in-field extraction method was used for water and soil matrices. A coiled microextraction (CME) device was utilised for the introduction of the extracted samples into a portable gas chromatography–mass spectrometry (GC–MS) for analysis. The total combined extraction and analysis time was approximately 6.5 min per sample. Results demonstrated that the in-field extraction and analysis methods can screen for fifty-nine target organic contaminants, including polyaromatic hydrocarbons, monoaromatic hydrocarbons, phenols, phthalates, organophosphorus pesticides, and organochlorine pesticides. The method was also capable of tentatively identifying unknown compounds using library searches, significantly expanding the scope of the methods for the provision of intelligence at pollution incidents of an unknown nature, although a laboratory-based method was able to provide more information due to the higher sensitivity achievable. The methods were evaluated using authentic casework samples and were found to be fit-for-purpose for providing rapid in-field intelligence at pollution incidents. The fact that the in-field methods target the same compounds as the laboratory-based methods provides the added benefit that the in-field results can assist in sample triaging upon submission to the laboratory for quantitation and confirmatory analysis.

**Supplementary Information:**

The online version contains supplementary material available at 10.1007/s11356-023-28648-w.

## Introduction

First responders to environmental incidents aim to protect the environment and human health (NSWEPA 2020). Field investigators rely on results obtained by a reach-back laboratory to manage the polluted site (Lam et al. 2019a; Spikmans 2015). However, in rapidly unfolding emergency response scenarios, these results are not typically available whilst the site investigation is still taking place, as the turnaround times of the reach-back laboratory are too long (Kaljurand 2014; Kalnicky and Singhvi 2001; Lam et al. 2018). The field officers are therefore reliant on their investigative skills to best protect the environment, without the privilege of having access to accurate scientific data on the pollutant(s) present (Lam et al. 2018; Spikmans 2015). This issue has been explained in past literature, and suggestions have been made to overcome this by using field-portable analytical equipment. This equipment can provide intelligence at the scene of the incident, whilst the incident is being investigated (Galuszka et al. 2015; Lam et al. 2018; Spikmans 2019; Turl and Wood 2008; Valcárcel and Cárdenas 2005).

The in-field results do not need to be confirmatory and do not have to withstand scrutiny in court (Lam et al. 2019b; Spikmans 2019). The in-field results need to provide preliminary information on the pollutant(s) present in a timely manner to allow the field officer to manage the site to protect the environment (Galuszka et al. 2015; Lam et al. 2018; Spikmans 2019). Ideally, the results are sufficiently characteristic to provide a chemical profile to aid in source-tracking (Kalnicky and Singhvi 2001; Spikmans 2019; Turl and Wood 2008; Valcárcel and Cárdenas 2005). Qualitative results (pollutant identification) are more important than quantitative results (the concentration of the pollutant), with the in-field methods only needing to provide a general indication of concentration (low, or high). Laboratory-based results are then used to confirm the in-field findings and to quantitate the pollutants present to allow the regulatory aspect of the investigation to take place (Spikmans 2019; Valcárcel and Cárdenas 2005).

Given these requirements, miniaturised field-portable equipment is highly suited for the task (Guidotti et al. 2020; Lam et al. 2019b; Valcárcel and Cárdenas 2005). Fit-for-purpose results can be obtained despite potential sacrifices in performance due to miniaturisation, shorter analysis times, and whilst being used in uncontrolled environmental conditions (Bartley et al. 2007; Galuszka et al. 2015; Turl and Wood 2008). Consequently, full validation studies, as are performed in laboratory settings, are not required, and the established and evaluated in-field methods need to be fit-for-purpose (Bartley et al. 2007; Spikmans 2019).

Due to the complex nature of the samples that are commonly encountered during environmental investigations (Cattle et al. 2004; Kaljurand 2014; Spikmans 2015), methods that are developed for in-field application should not only be capable of detecting common target compounds in a range of matrices but should also be capable of screening and identifying non-target compounds using library searches (Galuszka et al. 2015; Lam et al. 2019a). This would provide for a generic approach and would generate sufficient information to guide the on-site investigation until confirmatory results are provided by the reach-back laboratory (Lam et al. 2019b; Valcárcel and Cárdenas 2005).

The aim of this study was therefore to develop a rapid, field-based, qualitative analysis protocol for organic pollutants using a portable GC–MS. The method was designed to identify not only target compounds that are commonly detected by environmental laboratories but also non-target compounds. By using a similar approach to the laboratory, the in-field results can be used by the laboratory for the triaging of samples and to guide their analysis pathway.

A rapid portable GC–MS method was combined with rapid, in-field extraction methods for water and soil matrices, with the specific purpose of providing intelligence in the field in a timely manner. The methods were, therefore, aimed at emergency response scenarios and routine casework that are either unfolding quickly or are likely to have a relatively short site investigation, where rapid intelligence would be of most benefit (Mudge 2008; Spikmans 2019). Although several other studies have developed methods for the detection and identification of organic pollutants using a portable GC–MS, these methods are either not generic in the sense that the methods have not been shown to be able to screen for an array of pollutants, have not been demonstrated to be suitable for a range of matrices, and/or the methods are not as rapid as those discussed here (Hu et al. 2019; Truong et al. 2016, 2017; Wang et al. 2021; Zhang et al. 2016).

## Materials and methods

### Materials

Filters (PTFE, 13 mm, 0.22 µm) were obtained from Phase Separations, Australia. Anhydrous sodium sulphate powder, HPLC grade dichloromethane, and HPLC grade hexane were obtained from Sigma-Aldrich, Australia. The analytical standards used in this study were prepared from the following solutions: a custom semi-volatile organic compound mix (PM Separations, Australia), a polyaromatic hydrocarbon (PAH) organic compound mix (Trajan Scientific, Australia), a ‘single column analytes’ mix (Novachem, Australia), and an 8270 phenols standard (Phenomenex, Australia). Phenol-d6, phenanthrene-d10, 1,4-dichlorobenzene-d4, acenaphthene-d10, p-terphenyl-d14, and chrysene-d12 were used as internal standards and were obtained from Sigma-Aldrich, Australia.

### Standards, matrices, and casework samples

The analytical standards used in this study ranged in concentration from 0.15 to 10 ppm and contained seventy organic compounds that are commonly tested for by operational environmental laboratories using GC–MS. These compounds included PAHs, monoaromatic hydrocarbons (MAHs), phenols, phthalates, organophosphorus pesticides (OPs), and organochlorine pesticides (OCs). These standards were prepared in dichloromethane (DCM) using the four organic compound mixes described above as well as the six internal standards. The compounds used in this study are potentially hazardous to human health, and users are to consult safety data sheets prior to using these compounds.

Three soil matrices (soil 1, soil 2, and blank sand) were used during the method development. Soil 1 contained an organic matter content of 0.0118% (% loss on ignition or %LOI) and moisture content of 2.3%, and was collected from the general farm area of the NSW Department of Planning and Environment (DPE) ‘Centre for Recycled Organics in Agriculture’, at Belgenny Farm in Camden, NSW, Australia (Woodward 2018). Soil 2 contained a %LOI of 0.0081% and moisture content of 7.1%, and was collected from an area near the Jenolan Caves, Jenolan, NSW, Australia (Woodward 2018). The two soil matrices were selected to represent matrices of varying organic and moisture contents that could be encountered at pollution incidents in NSW, Australia. The blank sand was coarse sand purchased from a local hardware store that had been sifted to remove the > 2 mm fraction. The sand was then heated in a furnace at 400 °C, rinsed with DCM followed by drying to remove all organic material (USEPA 2007a). The sand was prepared and analysed by GC–MS, to ensure that no organic material remained. The sand contained no organic pollutants, organic matter, and moisture content and was used for quality control purposes.

Fourteen different real-world river water matrices collected from around the Sydney and Blue Mountains area, NSW Australia, were also used during the method development.

Authentic case samples (fifty soil samples and six water samples) were used to test the application of the developed method and were provided by the Department of Planning and Environment (DPE). Two of the water samples provided were authentic water runoff samples that were analysed as part of a mock pollution incident investigation.

### Torion T-9 Portable GC–MS

The portable GC–MS utilised in this study was a Torion Tridion-9 (Perkin Elmer, Inc., US), that consisted of a low thermal mass capillary GC with a miniaturised toroidal ion trap mass spectrometer. The instrument uses a 5-m MXT-5 Crossbond diphenyl dimethyl polysiloxane (0.1 mm ID × 0.4 µm film thickness) column and was capable of fast turnaround times of approximately 5 min, not including sample collection and preparation times (Smith et al. 2005).

Performance validation checks, using a CALION Performance Validation (PV) mix (Perkin Elmer, Inc., USA), were performed at the start of each analysis day to ensure that the GC–MS instrument was operating to specifications. System and sampling accessory blanks were also performed at the start and end of each test day, and throughout sample analysis to monitor for any potential cross-contamination between samples.

An external laptop was used to process the data using the CHROMION version 1.2.0.8 software (Perkin Elmer, Inc., USA). Compound identifications were made using the instrument’s on-board target library, which contained mass spectra added by the instrument’s manufacturer, as well as a purpose-built target compound library developed in this study. Any peaks that could not be identified via the on-board libraries were further evaluated using the NIST 2014 mass spectral library (version 2.2) (NIST, US).

Both soil and water matrices were extracted (refer below) and introduced into the portable GC–MS using a coiled microextraction (CME) device (Perkin Elmer, Inc., USA), which consisted of a fine, tightly wound stainless steel coil, designed for sampling liquids (Truong et al. 2017). The CME was loaded with three 3.5 µL drops of extraction solution, allowing the solvent to evaporate completely between each applied drop and before injection into the instrument (Truong et al 2017).

The portable GC–MS injection port was 270 °C with a desorption time of 5 s. The system was set to splitless injection. The column temperature started at 50 °C which was held for 10 s before increasing at 2 °C per second to a final temperature of 285 °C. The final temperature was held for 60 s, resulting in a total analysis time of 187.5 s (3.1 min). The transfer line between the GC column and the MS detector was set to 250 °C. A solvent delay of 40 s was used, and the MS was set to record the mass range of 43 to 500 amu.

### Field-based soil and water extraction method

The extraction method used was based on the method provided by Truong et al. (2016). For soil matrices, 2 mL of 1:1 DCM/hexane was added to approximately 1 mL of soil. The soil was homogenised using a spatula, and large stones and other extraneous items, such as sticks, were removed before extraction. For water matrices, 2 mL of DCM was added to 10 mL of water. The extraction process was the same for both matrix types. The sample (soil or water) with solvent was shaken for 30 s. The solvent/extract layer was removed and placed on ~ 0.5 g of anhydrous sodium sulphate to remove any residual water. The dried extract was then filtered through a 0.45-µL PTFE syringe filter ready for CME sampling.

### Laboratory-based GC–MS

The results of the portable GC–MS were compared to those obtained by an Agilent 6890/5973 benchtop GC–MS (Agilent Technologies Australia Pty Ltd, Australia). The instrument used a 30-m Agilent J&W DB-5MS Ultra Inert column (0.25 mm × 0.25 µm film thickness). The column temperature started at 40 °C and increased at 10 °C per minute until it reached 300 °C where it was held for an additional 7 min, giving a runtime of 35 min. The injection port of the laboratory GC–MS was 250 °C with an injection volume of 2.0 µL. The system was set to pulsed splitless with a pulse time of 1 min and a pulse pressure of 16 psi. The transfer line between the GC column and the MS detector was 280 °C. The MS had a solvent delay of 6.5 min and was set to record the mass range of 35 to 550 amu.

### Method evaluation relative to the laboratory method

The field-based method (extraction and analysis combined) was evaluated against the laboratory-based method for the qualitative analysis of target compounds. The evaluation was performed based on Technical Note 17 of the National Association of Testing Authorities (NATA), Australia (2013), and the publication by Fiorentin et al. (2020).

True positive (TP), true negative (TN), false positive (FP), and false negative (FN) results were determined by comparing the compound identification from the field-based method to the laboratory-based results. The sensitivity, specificity, accuracy, positive predictive value (PPV), and negative predictive value (NPV) were calculated as percentages using the following equations (NATA 2013; Fiorentin et al. 2020):

$$\begin{array}{l}\mathrm{Sensitivity}:100\times \left(\mathrm{TP}\right)/\left(\mathrm{TP}+\mathrm{FN}\right)\\ \mathrm{Specificity}:100\times \mathrm{(TN)}/\left(\mathrm{TN}+\mathrm{FP}\right)\\ \begin{array}{l}\mathrm{Accuracy}:100\times \left(\mathrm{TP}+\mathrm{TN}\right)/\left(\mathrm{TP}+\mathrm{TN}+\mathrm{FP}+\mathrm{FN}\right)\\ \begin{array}{l}\mathrm{Positive predictive value }(\mathrm{PPV}):100\times \left(\mathrm{TP}\right)/\left(\mathrm{TP}+\mathrm{FP}\right)\\ \mathrm{Negative predictive value (NPV}):100\times \left(\mathrm{TN}\right)/\left(\mathrm{FN}+\mathrm{TN}\right)\end{array}\end{array}\end{array}$$

## Results and discussion

### Validation requirements for field-based methods

As previously discussed, the use of person-portable equipment is not intended to replace laboratory analysis to initiate regulation and court proceedings, but rather to obtain in-field intelligence to guide the management of the polluted site to protect the environment. In-field methods based on person-portable equipment therefore do not need to be validated to the same level of rigour as laboratory-based methods.

Contrary to environmental monitoring, concentrations of pollutants are expected to be high in emergency response scenarios where the incident has recently occurred. An emergency response scenario requires fast analysis times, where the priority is to inform first responders within a timely manner what pollutants are present. The most important type of intelligence that is required is therefore qualitative in nature (identification of target and non-target compounds that are present) (Bartley et al. 2007; Galuszka et al. 2015; Guidotti et al. 2020). The time required to obtain quantitative results in the field using a portable GC–MS outweighs the potential benefits.

To validate the portable GC–MS methods developed in this study, the detection limits, accuracy, and repeatability of the methods were determined (NATA 2013). Detection limits needed to be established to provide the operator with an understanding of when the methods are suitable for the scenario at hand and their limitations. If concentrations of pollutants are at the detection limit, then the operator must be aware of the potential for false negative results. Accuracy refers to the correct identification of compounds with repeatability referring to correct identification of compounds on multiple occasions. In addition, matrix interference was evaluated by analysing authentic samples and by exposing the methods to field conditions. Comparisons were made against results from a laboratory-based GC–MS method (operated by one of the co-authors for their routine environmental analysis work using methods based on USEPA 2018) for evaluative purposes for both target and non-target compound detection and identification. The aim was to determine whether the portable GC–MS could correctly identify the target and non-target compounds present.

### Development of soil and water extraction methods for field application

Within the laboratory, samples typically undergo a lengthy solvent extraction process. For soils, this can involve grinding and drying the sample (USEPA 1994, 2007b), pressurised solvent extraction, QuEChERS, and/or sonication. For water samples, it involves using liquid–liquid extraction with a suitable solvent (USEPA 2007b). Although these processes allow for high-yield extraction of the organic compounds present, as well as concentrating them up to improve detection limits, they are too time consuming for field application and require equipment that cannot be utilised in the field. It was therefore necessary to develop an extraction method suitable for in-field use. This extraction method needed to consider a range of factors, including sample amount, the use of solvents in the field, and the safety of the operator (as a fume hood will likely not be available in most instances). Ideally, the method was very similar for soil and water matrices to simplify field operations and to reduce the amount of chemicals and materials required to be carried. This method ideally needed to be simple and quick to perform to be suitable for emergency response scenarios, whilst providing reliable qualitative information.

The final developed extraction methods for both soil and water matrices follow the same procedure (refer to material and method section), with the only difference being the amount of sample and type of solvent used. The methods were based on those developed by Truong et al. (2016). The entire extraction process takes approximately 1.5–2 min to perform, creating a total sampling time of approximately 3 min per sample when loading the CME three times prior to analysis. The method uses small volumes of organic solvents and can be performed in the field using appropriate PPE (including a suitable respirator) if a fume hood is not available (such as in a mobile laboratory).

Although soil samples are usually weighed before extraction, the developed field method used the volume of the soil as an approximate measure. Using volumes avoids the need to carry a balance into the field, and the method is suitable for qualitative analysis. The soil extraction method was developed for 1 mL of soil to minimise the amount of solvent needed and therefore the overall amount of waste that is to be carried back out of the field for appropriate disposal. Although moisture content is not considered, this is not critical for a qualitative method.

Taking a small amount of soil for extraction can introduce some variability in results due to the inhomogeneity of soil. Nevertheless, considering the requirement of speed and simplicity and the focus on qualitative results rather than determining accurate concentration of pollutants present, 1 mL of soil was deemed adequate. A minimum of 2 mL of solvent was required; otherwise, it was too difficult to pipette the solvent from the soil during extraction. This does reduce the sample concentration, but the extraction method was still found to be suitable as will be discussed below. This approach was preferred over filtering the samples as filtering would require more equipment to be transported into the field and would result in longer sample preparation times.

For the water extraction method, 10 mL of water was extracted using 2 mL of DCM, allowing the sample to be concentrated by a factor of five to improve detection levels, whilst keeping the amount of solvent that needs to be transported and used in the field to a minimum.

The combined preparation (3 min) and analysis time (3.5 min) for soil and water samples were approximately 6.5 min. Compared to standard laboratory methods that typically take ca. 1 to 2 h per sample, this is a significant improvement in turnaround time and was deemed suitable for in-field use.

### Development of a target compound library

The portable GC–MS utilises a miniaturised toroidal ion trap, which can produce differences in mass ion arrangements when compared to a quadrupole MS. The difference in mass spectrometer may produce lower match probabilities against a NIST library, or at least requires user input to check the library results. To improve data processing times, as well as the accuracy of the compound identifications, it was necessary to develop a personal library containing the retention times and mass ions of target compounds. The analysis of unknown compounds that are observed during any screening process still required comparison against additional libraries for tentative identification.

Analytical standards that contained 70 semi-volatile organic compounds that are commonly screened for by operational environmental laboratories using GC–MS were analysed to create the personal library. These compounds are based on the USEPA 8270E method: semivolatile organic compounds by gas chromatography/mass spectrometry (USEPA 2018).

**Fig. 1:**
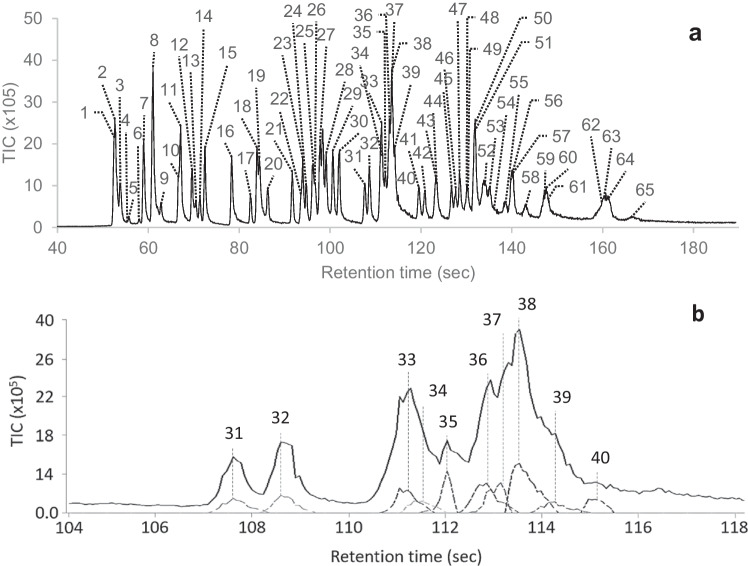
Representative total ion chromatogram of a 5-ppm standard analysed on the portable GC–MS using CME sampling. **a** Compounds identified within the standard and **b** an example of the deconvolution peaks for compounds 31 to 40 that are generated by the instrument’s deconvolution software. Refer to Table 1 for compound identifications

Representative results obtained for a 5-ppm standard are provided in Fig. 1A and Table 1. These results portray that within just a 3.5-min analysis, the portable GC–MS could detect fifty-nine of the organic target compounds as well as the six internal standards. This is impressive considering that the laboratory method can take up to 40 min per analysis. The rapid analysis time, however, does have a trade-off, with reduced separation being observed for many of the target compounds.

**Table 1:** Target compounds that are identifiable by the portable GC–MS, including their retention times. The quantitation and qualifier ions used to confirm their identity are also included for each compound. Compounds with an asterisk are internal standards

Compound name	Ret time (sec)	Quant ion (m/z)	Qual ions (m/z)	Compound name	Ret time (sec)	Quant ion (m/z)	Qual ions (m/z)	Compound name	Ret time (sec)	Quant ion (m/z)	Qual ions (m/z)
1. Phenol	52.6	94	65 66	23. Acenaphthene	94.0	153	154 152	44. Aldrin	123.4	66	261 263
2. Phenol-d6*	52.7	99	71	24. 2,4‐ Dinitrophenol	94.8	184	63 154	46. Isodrin	126.8	193	195 66
3. 2‐ Chlorophenol	53.9	128	64 130	25 Pentachlorobenzene	96.3	250	252 108	47. Heptachlor Epoxide	127.6	353	355 351
4. 1,4- Dichlorobenzene-d4*	55.8	150	152 115	26. 2,4‐ Dinitrotoluene	96.7	165	63 89	48. Fluoranthene	128.5	202	200 203
5. 1,4‐ Dichlorobenzene	55.8	146	148 111	27. 2,3,4,6‐Tetrachlorophenol	97.8	232	230 131	48. Gamma‐chlordane	131.7	373	375 377
6. 1,2‐ Dichlorobenzene	58.1	146	111 75					49. Alpha‐chlordane	132.0	373	375 377
7. 2‐ Methylphenol (o-cresol)	59.0	108	107 79	28. 2,3,5,6-Tetrachlorophenol	99.1	198	196 126	50. Endosulfan I	132.4	195	237 339
8. 3 + 4‐ Methylphenol (m + p-cresol)	61.0	107	108 77	39. Fluorene	100.6	166	165 163	51. Pyrene	133.0	202	200 203
9. Nitrobenzene	62.8	77	123 51	30. 2‐Methyl‐4 6‐dinitrophenol	101.9	198	121 105	52. p,p′‐DDE	133.8	246	248 318
10. 2‐Nitrophenol	66.7	139	109 65	31. Alpha‐BHC	107.6	181	183 219	53. Dieldrin	135.0	79	263 277
11. 2,4‐Dimethylphenol	67.1	122	**107** 121	32. Hexachlorobenzene	109.5	284	286 288	54. Terphenyl-d14*	136.0	244	245 122
12. 2,4‐Dichlorophenol	69.7	162	164 98	33. Pentachlorophenol	111.3	266	264 268	55. Endrin	138.7	81	263 265
13. 1,2,4‐Trichlorobenzene	70.5	180	145 109	34. Beta/Gamma‐BHC	111.6	181	183 219	56. p,p′‐DDD	139.9	235	237 165
14. Naphthalene	71.3	128	126 102	35. Pentachloronitrobenzene	112.2	237	214 142	57. Endosulfan II	140.2	195	241 339
15. 2,6‐Dichlorophenol	72.5	162	164 98	36. Phenanthrene-d10*	113.0	188	80 94	58. Endrin aldehyde	143.2	67	345 250
16. 4‐Chloro‐3‐methylphenol	78.3	107	144 142	37. Phenanthrene	113.3	178	176 152	59. p,p′‐DDT	147.1	235	165 237
17. 1,2,4,5‐Tetrachlorobenzene	82.5	216	214 179	38. Dinoseb	113.7	211	163 147	60. Endosulfan sulphate	147.4	272	274 387
18. 2,4,6‐Trichlorophenol	83.4	196	198 200	39. Anthracene	115.0	178	176 152	61. Bis(2‐ethylhexyl) adipate	148.2	129	112 147
19. 2,4,5‐Trichlorophenol	84.4	196	198 97	40. Delta‐BHC	115.7	181	183 219	62. Benzo (a) anthracene	160.1	228	226 229
20. 1,2,3,4‐Tetrachlorobenzene	86.3	216	214 218	41. Heptachlor	119.6	100	272 274	63. Chrysene-d12*	160.7	240	120 236
21. Acenaphthylene	91.7	152	150 151	42. Dibutyl phthalate	120.9	149	150 223	64. Chrysene	161.5	228	226 229
22. Acenapthene-d10*	93.6	162	164 160	43. Chlorpyrifos	123.2	199	197 314	65. Bis(2‐ethylhexyl) phthalate	166.4	149	167 279

As shown in Fig. 1B, the instrument’s CHROMION deconvolution software can provide some compensation for reduced chromatographic performance by identifying the presence of compounds based on the mass ions present within overlapping peaks (Truong et al. 2016). The effectiveness of the deconvolution, however, can be impacted by the number of compounds present within the overlapped peak. It is unlikely that a real-world sample would be encountered that contains such a high number of target compounds; however, by analysing this standard, it allowed for an initial assessment of the method’s selectivity for the target compounds that elute at similar retention times. Overall, it was found that the portable GC–MS could provide relatively easy identification for all but eleven of the target compounds and that this level of separation was deemed suitable for in-field use in favour of the 3.5-min analysis time.

The same quantification and qualifier ions as used by an equivalent laboratory-based GC–MS method were used to confirm the identity of the target compounds, along with the elution order. Note that quantification was not performed on the portable GC–MS, but the term ‘quantification ion’ was used to allow translation to laboratory-based methods. The associated spectra and retention times for each compound were used to create a target library on the portable GC–MS (see Table 1).

Eleven target compounds were not detected but these were mainly heavy compounds that would have eluted after 170 s, including methoxychlor, endrin ketone, and the heavy PAHs benzo(a)pyrene, benzo(b)fluoranthene, benzo(ghi)perylene, benzo(k)fluoranthene, dibenzo(ah)anthracene, indeno(1,2,3-cd) pyrene, and perylene. Despite adjusting GC method parameters, including higher injector and column temperatures, the instrument’s sensitivity significantly decreases after approximately 150 s. The method could therefore provide false-negative results for these heavy compounds, and this needs to be taken into consideration when deploying the method in the field. Only one compound that eluted earlier was not detected (4-nitrophenol). It is unclear why this compound was not detected, but this is likely related to resolution issues.

### Determining instrument sensitivity for target compounds

Although the methods developed in this study are for qualitative screening, rather than quantitation, five repeat analyses of a 5-ppm and seven-repeat analyses of each of the 0.5-ppm and 0.15-ppm standards were performed to estimate the instrument detection limit (IDL) and determine if the instrument is likely to be sufficiently sensitive for the application. The laboratory-based methods operated by the co-authors use the 0.15-ppm standard as the lowest calibration standard for routine analysis.

Most of the compounds in these standards were present at the concentration indicated, but some compounds within the mixture were present at slightly higher concentrations. For instance, in the 0.5-ppm standard, most compounds were present at 0.5 ppm, but some compounds were present at different concentrations up to 4 ppm (refer to Supplementary Information, Tables S1 – S3). The compounds were mixed at these different concentrations as they provided a similar response factor in the laboratory-based GC–MS at these different concentrations.

By loading the CME with three 3.5 µL drops, the portable GC–MS could detect compounds down to 0.5 ppm with identifications made using a combination of the developed automatic peak detection and target library search, and a search using extracted ions. To detect compounds listed in Table 1 within the lowest standard of 0.15 ppm, the CME had to be loaded with six 3.5 µL drops, and even then, not all compounds could be detected repeatably. Although it is possible to add more drops to the CME to increase sensitivity, for ease of field use, and to reduce sample preparation times, the IDL was determined with only three drops of solution being added to the CME. The ability of the method to accept different amounts of samples based on the number of times the CME is loaded demonstrates the robustness of the method.

The IDL was therefore determined to be with the 0.5-ppm standard as the compounds were all detected in all seven replicates, except for 2-chlorophenol, 1,2-dichlorobenzene, 1,4-dichlorobenzene, 2-nitrophenol, 2,4-dinitrophenol, and 1,2,4-trichlorobenzene. These compounds were sometimes, but not always, detected in the 0.5-ppm standard, and the IDL for these compounds is therefore an estimation. Table S2 in the Supplementary Information provides the IDL concentration for the individual compounds.

**Fig. 2:**
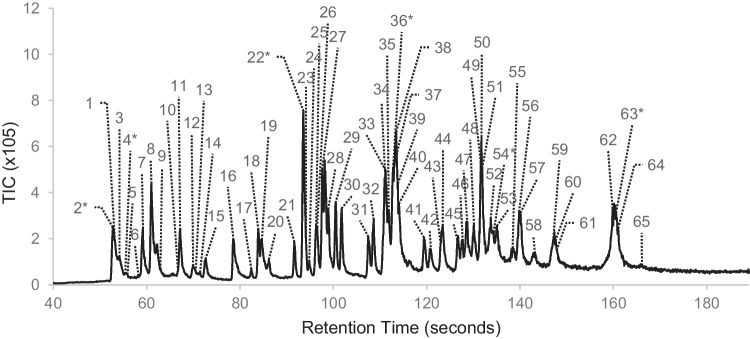
Representative total ion chromatogram of a 0.5-ppm standard analysed on the portable GC–MS using CME. The internal standards, labelled compounds 2, 4, 22, 36, 54, and 63, are present at ~ 2 ppm in each standard and are indicated with an asterisk. Refer to Table 1 for the compound identifications

Figure 2 provides a representative chromatogram for one of the 0.5-ppm replicates that was able to detect all the target compounds listed in Table 1. As previously explained, the compounds present in this standard ranged in concentration from 0.5 to 4 ppm; the IDL is, therefore, in this range of concentrations.

Overall, the portable GC–MS is sensitive enough to detect compounds at a concentration close to that of the lowest standard analysed by the laboratory-based method. However, it is to be noted that this is when analysing the standards directly, without an extraction process, potential matrix interferences, and whilst operating under laboratory conditions. As previously mentioned, the laboratory-based extraction method is designed to pre-concentrate the sample prior to analysis, and it is therefore expected that the field-based method will have a higher method detection limit (MDL).

### Evaluation of the soil and water extraction methods using matrix spike samples

It was important to provide an estimate of the MDL of the target compounds within different soil and water matrices to demonstrate the applicability of the field-based extraction methods for emergency response applications. To determine the MDL, three different soil matrices and fourteen different river water matrices were prepared and spiked at two concentrations close to the IDL. Both the soil and water matrices had previously been analysed and determined to not contain any of the target compounds and were therefore suitable to be used as a blank matrix.

A 10-ppm analytical standard containing target compounds ranging from 10 to 80 ppm was used to spike the samples. Soil samples were prepared at two different concentrations by spiking 100 and 200 µL of the 10-ppm analytical standard into 1 mL of each soil. Because the particle density of the used soils is approximately 2.1 g/cm^3^ (Woodward 2018), the concentration in w/w (dried) would be approximately 0.5–4 ppm (low spiked soil) and 1–8 ppm (high spiked soil) respectively. Although there is a slight error in this calculation due to moisture content (moisture content ranged from 0 to 7% (Woodward 2018)), the concentration of pollutants in emergency incidents is generally significantly higher as will be explained below. It was therefore not critical to determine the exact MDL level and an estimated MDL was considered suitable.

Fourteen different river water matrices were prepared by spiking 10 mL of the selected water matrix with 200 µL of the 10-ppm standard, to provide a concentration of 0.2–1.6 ppm (low spiked water). In addition, a high spiked water sample was prepared using one of the river water matrices by spiking 400 µL of the 10-ppm standard into 10 mL of water, giving a concentration of 0.4–3.2 ppm.

Seven replicates were prepared and analysed for each soil matrix (low and high spiked soil) and the high spiked water. The fourteen different river samples were only prepared and analysed once each. The target compounds were screened for using a combination of the automatic peak detection and target library search, and manual extracted ion search. The CME was loaded with three 3.5 µL drops for each sample analysed.

Table 2 provides the estimated MDLs for the target compounds in soil and water matrices. The internal standards have been excluded from this table for simplification. The MDLs were determined based on whether that compound could be repeatably detected at the indicated concentration across the three soil matrices and the fourteen river samples (refer to Supplementary Information – Tables S4–S8).

**Table 2:** Estimated method detection limits (MDL) for target compounds within soil and water matrices when using the developed soil/water extraction methods followed by analysis on the portable GC–MS using CME sampling. The MDLs were determined from seven replicate analyses of three soil matrices, seven replicate analyses of a river water sample, and analysis of fourteen river water matrices

Target compound	MDL (ppm)		Target compound	MDL (ppm)	
	Soil	Water		Soil	Water
Phenol	2	1.6	Beta or Gamma‐BHC	0.5	0.2
2‐Chlorophenol	2	0.8	Pentachloronitrobenzene	0.5	0.2
1,4‐Dichlorobenzene	1	0.2	Phenanthrene	0.5	0.2
1,2‐Dichlorobenzene	1	0.2	Dinoseb	2	0.8
2‐Methylphenol (*o*-cresol)	2	0.8	Anthracene	0.5	0.2
3 + 4‐Methylphenol (*m* + *p*-cresol)	4	1.6	Delta‐BHC	0.5	0.2
Nitrobenzene	2	0.2	Heptachlor	0.5	0.2
2‐Nitrophenol	4	0.8	Dibutyl phthalate	0.5	0.2
2,4‐Dimethylphenol	2	0.8	Chlorpyrifos	1	0.2
2,4‐Dichlorophenol	2	0.8	Aldrin	1	0.2
1,2,4‐Trichlorobenzene	1	0.2	Isodrin	0.5	0.2
Naphthalene	1	0.2	Heptachlor epoxide	1	0.4
2,6‐Dichlorophenol	2	0.8	Fluoranthene	0.5	0.2
4‐Chloro‐3‐methylphenol	2	0.8	Gamma‐chlordane	1	0.4
1,2,4,5‐Tetrachlorobenzene	0.5	0.2	Alpha‐chlordane	1	0.4
2,4,6‐Trichlorophenol	2	0.8	Endosulfan I	2	0.8
2,4,5‐Trichlorophenol	2	0.8	Pyrene	0.5	0.2
1,2,3,4‐Tetrachlorobenzene	0.5	0.2	p,p′‐DDE	0.5	0.2
Acenaphthylene	0.5	0.2	Dieldrin	1	0.4
Acenaphthene	0.5	0.2	Endrin	> 1	> 0.4
2,4‐Dinitrophenol	> 4	> 1.6	p,p’‐DDD	0.5	0.2
Pentachlorobenzene	0.5	0.2	Endosulfan II	2	0.8
2,4‐Dinitrotoluene	1	0.2	Endrin aldehyde	> 1	> 0.4
2,3,4,6‐Tetrachlorophenol	2	0.8	p,p′‐DDT	1	0.4
2,3,5,6-Tetrachlorophenol	2	0.8	Endosulfan sulphate	2	0.8
Fluorene	0.5	0.2	Bis(2‐ethylhexyl) adipate	1	0.4
2‐Methyl‐4 6‐Dinitrophenol	> 4	> 1.6	Benzo (a) anthracene	0.5	0.2
Alpha‐BHC	0.5	0.2	Chrysene	0.5	0.2
Hexachlorobenzene	0.5	0.2	Bis(2‐ethylhexyl) phthalate	1	0.4
Pentachlorophenol	2	0.8			

Overall, most compounds eluting before 125 s could be reliably detected in all the lower spiked soil and water matrices. Many of the compounds that eluted after 125 s (except for PAHs and some OCs) could only be reliably detected in the higher spiked soil and water samples, resulting in higher MDLs for these compounds. The early eluting benzene compounds and naphthalene were also not repeatably detected in the lower spiked soil samples, also leading to higher MDLs for these compounds.

Both the soil and water extraction methods also showed low selectivity for the 2,4-dinitrophenol, 2‐methyl‐4 6‐dinitrophenol and two endrin compounds as they could not be repeatably detected across any of the spiked samples despite them being present at a higher concentration in relation to the other compounds. This appears to be a limitation of the developed extraction methods.

Except for the compounds mentioned, the water and soil methods demonstrated good repeatability and accuracy in detecting target compounds at an MDL range of 0.5–4 ppm within soil matrices and 0.2–1.6 ppm within water matrices.

These MDL results need to be considered in context. The aim of the developed methods was to use these for emergency response situations where a pollution incident is unfolding. The authors collectively have decades of real-world experience in investigating pollution incidents, and the concentrations of pollutants of concern at an emergency response are generally going to be significantly higher than the concentrations indicated for IDL and MDL. Even though the in-field methods are not as sensitive as the laboratory methods, due to the inability to further concentrate samples in the field and limitations associated with instrument performance, the focus of an emergency response is to manage the site rather than conduct environmental monitoring and for this purpose the sensitivity of the methods is considered adequate.

### Application to real-world casework samples for target compound detection

A range of authentic casework samples were analysed to evaluate the developed in-field methods against laboratory-based methods for the correct detection and identification of target compounds. Fifty soil samples and six water samples were analysed, chosen based on availability of authentic samples at the time of conducting this study, whilst containing target compounds at or above the established MDLs. It should be noted that not all samples were originally collected from pollution incidents and most samples were collected for the purpose of environmental monitoring. Nevertheless, these samples were considered suitable to evaluate the developed methods. The samples were analysed and interpreted with no prior knowledge of the laboratory-based results, where the laboratory results were only shared after all analyses on the portable GC–MS were completed and reported.

**Fig. 3:**
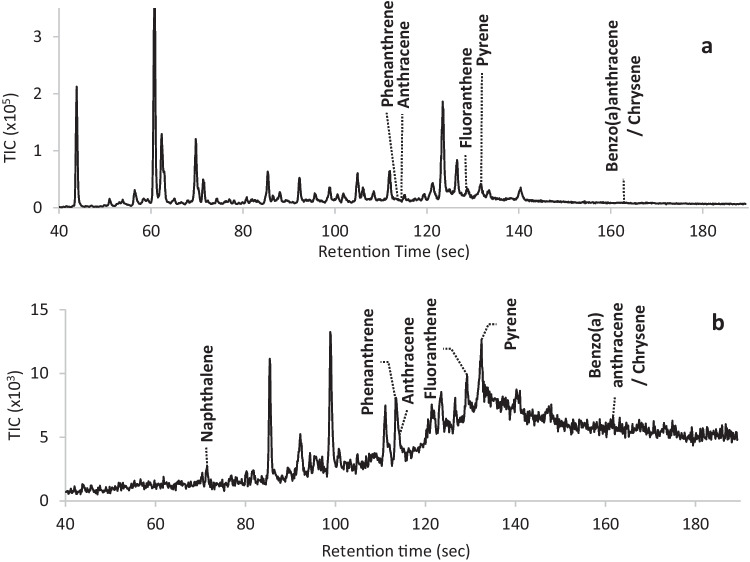
Representative total ion chromatograms obtained for two authentic soil casework samples (**a** and **b**) analysed on the portable GC–MS with CME sampling. The target compounds detected are indicated. Some of the target compounds did not generate a visible peak in the total ion chromatogram and were detected via an extracted ion search

Figure 3 provides two representative chromatograms (A and B) of the soil samples analysed. The identified target compounds are indicated in the figure. As would be expected with soil samples, both chromatograms demonstrate that many non-target compounds were detected, allowing the methods to be evaluated for matrix interferences. Due to the various non-target compounds present, it was found that although the deconvolution software was able to detect many target compounds using the target library search, it could not detect all that were present, and manual extracted ion searches were required to ensure further target compounds were detected and identified. Extracted ion searches were therefore performed on all samples alongside the automated peak detection and target library search.

Table 3 provides an overview of the results obtained for the detection of target compounds in the fifty real-world soil samples (refer also to Supplementary Information – Table S9). The results are compared to those obtained by the laboratory-based method. The concentration range of the target compounds that were detected based on dried weight by the laboratory-based method is also provided. No false positive results were obtained using the field-based method, but there were a number of false negative results recorded which affected the sensitivity values. It was found that most of the false negatives observed were due to matrix interferences, akin with that observed for Fig. 3A, whilst the compounds were present close to the MDL. The accuracy of the method was shown to be acceptable with values of 90% and above, except for anthracene as will be discussed below.

**Table 3:** Comparison of portable GC–MS results to the laboratory-based results for the detection and identification of target compounds in 50 authentic soil casework samples. Abbreviations and equations are provided in the methods section (NATA 2013; Fiorentin et. al. 2020). The concentration of the compounds present was determined by the laboratory-based method

*n* = 50	Soils									
Target compounds detected	Concentration range (ppm)	TP	TN	FN	FP	Accuracy (%)	Sensitivity (%)	Specificity (%)	PPV (%)	NPV (%)
Naphthalene	2.4–2.6	1	48	1	0	98	50	100	100	98
Fluorene	0.51–1.1	1	47	2	0	96	33	100	100	96
Acenaphthene	0.46–0.78	2	48	0	0	100	100	100	100	100
Phenanthrene	0.58–6.4	33	12	5	0	90	87	100	100	71
Anthracene	0.56–1.9	7	34	9	0	82	44	100	100	79
Fluoranthene	0.50–15	46	2	2	0	96	96	100	100	50
Pyrene	0.50–16	44	4	2	0	96	96	100	100	67
Benzo(a)anthracene/chrysene (values with increased MDL to 1 ppm)	0.50–7.3	31 (42)	4 (4)	15 (4)	0 (0)	70 (92)	67 (91)	100 (100)	100 (100)	21 (50)

Benzo(a)anthracene/chrysene produced the greatest number of false negative results across the fifty samples analysed. Based on the data obtained, it appears that the MDL determined in Table 2 might have been too low. If the MDL is increased from 0.5 to 1.0 ppm, the false negatives reduce from fifteen to four, which improved the sensitivity and accuracy towards these compounds (refer to Table 3). Hence, the MDL for these compounds is more likely to be around 1.0 ppm.

Anthracene produced the second greatest number of false negatives. Further evaluation of the chromatograms found that there was interference between anthracene and phenanthrene whenever phenanthrene was present at a greater concentration than anthracene. This is due to their similar retention times (only 2 s apart) where the peaks were not resolved due to the compressed run time, as well as the same ions being used to identify both compounds. The presence of anthracene, therefore, cannot be ruled out whenever phenanthrene is detected, and this is reflected in the accuracy shown in Table 3. Nevertheless, if either compound is detected, the field officer is provided with information that PAHs are present.

**Fig. 4:**
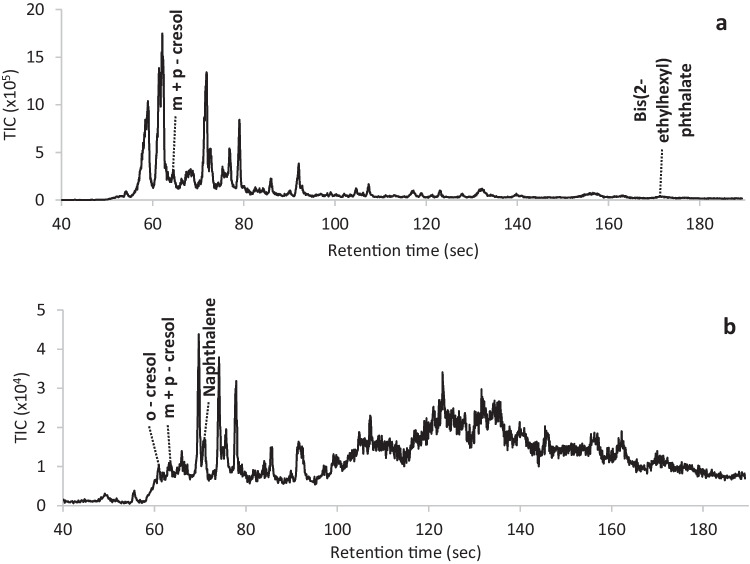
Representative total ion chromatograms obtained for two authentic water casework samples (**a** and **b**) analysed on the portable GC–MS using CME sampling. The target compounds detected are identified. Some of the target compounds did not generate a visible peak and were detected via an extracted ion search

Figure 4 provides two representative chromatograms obtained for water matrices. Like the real-world soil samples, many non-target compounds can be observed.

Table 4 provides the results of the target compounds detected by the portable GC–MS within the six casework water samples (refer also to Supplementary Information – Table S10). Again, the results are compared to those obtained by the laboratory-based method. The portable GC–MS produced no false positives and one false negative for the presence of naphthalene despite it being present above the MDL. Visual examination of the chromatogram indicated that matrix interference was the cause. For the other target compounds that were present, the portable GC–MS was successful in indicating their presence as reflected by the accuracy values.

**Table 4:** Comparison of portable GC–MS results to the laboratory-based results for the detection and identification of target compounds in six authentic water casework samples. Abbreviations and calculations are provided in the methods section (NATA 2013; Fiorentin et. al. 2020). The concentration of the compounds present was determined by the laboratory-based method

*n* = 6	Water									
Compound	Concentration range (ppm)	TP	TN	FN	FP	Accuracy (%)	Sensitivity (%)	Specificity (%)	PPV (%)	NPV (%)
2-Methylphenol	0.12	2	4	0	0	100	100	100	100	100
3 + 4-Methylphenol	0.22–21	4	2	0	0	100	100	100	100	100
Naphthalene	0.12–80	2	3	1	0	83	67	100	100	75
2,4-Dimethylphenol	0.06	2	4	0	0	100	100	100	100	100
Bis(2-ethylhexyl) phthalate	24	1	5	0	0	100	100	100	100	100

Considering that only a small portion of each sample is analysed in comparison to standard laboratory-based extraction methods (often 10 g for soil and 100 mL for water), as well as no pre-concentration step being performed, the portable GC–MS was able to successfully indicate the presence of most of the target compounds within the soil and water samples that were present above the MDL. Although the MDL might be slightly higher than indicated in Table 2 for a few compounds, depending on the matrix interference present, the MDL is considered sufficiently low for pollution incidents. Because pollutants are generally present significantly above the MDL at emergency response incidents, matrix interference is unlikely to be an issue, demonstrating the potential for incorporating the method for in-field intelligence gathering.

### Application to real-world samples under field conditions at a mock pollution incident

A mock incident based on authentic casework samples was used to evaluate the developed extraction and portable GC–MS methods under field conditions. The mock trial mimicked a real-world callout to an incident where the samples were unknown to the analyst. The samples were extracted and analysed inside a mobile laboratory (Spikmans 2019), although the environmental conditions (temperature and humidity) were not controlled.

A field officer collected two water runoff samples (samples A and B) from a factory fire at a robotics/computer factory. The samples were submitted to the mobile laboratory for analysis with the request that the samples were screened for any potential organic contaminants.

The two samples were extracted using the water extraction method and analysed on the portable GC–MS using CME sampling.

The portable GC–MS results were processed by performing extracted ion searches alongside the automated peak detection and target library search. Non-target compounds were tentatively identified using the NIST 2014 database (NIST, US). Only those compounds obtained by the portable GC–MS method that were relevant to the scenario were reported.

Within 3 h of arriving on-site, a written report was provided to the field officer with the analytical results. The 3 h included obtaining the briefing from the field officers, writing out a sample receipt, setting up the mobile laboratory and the equipment, extracting and analysing the samples, generating and processing the data, packing up the mobile laboratory and the equipment, and writing the final analysis report. The results could be verbally provided to the field officer within 1–2 h of arriving on site.

The field officer was informed that both samples contained the target compounds 2-methylphenol, 3 + 4-methylphenol, and 2,4-dimethylphenol (Table 5). Both samples also appeared to contain a range of alkylated phenols, alkylated benzoic acid compounds, and some other benzene-based compounds.

**Table 5:** Portable GC–MS results of the target and non-target compounds detected within the fire runoff samples A and B that formed part of the mock scenario. Results reported by the laboratory-based method are also provided

	Sample A		Sample B	
	Portable GC–MS	Laboratory	Portable GC–MS	Laboratory
Target compounds	2-Methylphenol 3 + 4-Methylphenol 2,4-Dimethylphenol	2-Methylphenol 3 + 4-Methylphenol 2,4-Dimethylphenol Phenol	2-Methylphenol 3 + 4-Methylphenol 2,4-Dimethylphenol	2-Methylphenol 3 + 4-Methylphenol 2,4-Dimethylphenol Phenol
Non-target compounds (based on NIST library searches)	Methoxyphenol Ethylphenols Acetophenone Methylbenzoic acid Bisphenol Methoxy-benzeneamine Methylphenols	Methoxyphenol Ethylphenols Acetophenone Methylbenzoic acid Bisphenol Methylfurfural Isopropylphenol Hydroxymethylfurfural Triphenyl Phosphate	Methoxyphenol Ethylphenols Acetophenone Bisphenol Methoxy-benzeneamine Methylphenols	Methoxyphenol Ethylphenols Acetophenone Bisphenol Methylbenzoic acid Methylfurfural Isopropylphenol Hydroxymethylfurfural Triphenyl phosphate

Once the above result summary was provided to the field officer, the confirmatory results obtained by the laboratory were released for comparison. Table 5 provides a comparison of the compounds detected by the portable GC–MS and those reported by the laboratory.

The results provided in Table 5 show that, overall, the portable GC–MS was successful in providing accurate intelligence to the field officer. The method correctly indicated the presence of all the target compounds except for phenol, which was determined by the laboratory method to have been present at 0.98 ppm. This concentration is below the MDL for the portable GC–MS method, and with matrix interference present, it is not surprising that phenol was not detected.

The portable GC–MS was also capable of providing accurate identification for several non-target compounds that were also reported by the laboratory-based method. The laboratory-based method detected more non-target compounds than the portable GC–MS, but this is not unexpected given that the laboratory method is more sensitive.

Overall, the portable GC–MS performed well in the field during the mock scenario. Although it was not able to provide the same amount of information as the laboratory method, the portable GC–MS method was able to provide accurate intelligence within a timely manner that could have guided the incident management team on how to handle the incident site. The field-based method also provided presumptive results to the laboratory that could have guided how the samples could have been triaged for confirmatory analysis. Furthermore, the robustness of the method was demonstrated by exposing the method to uncontrolled environmental conditions.

## Conclusions

A fit-for-purpose, qualitative in-field sample extraction and analysis method based on a portable GC–MS using CME sampling was developed for the rapid detection and identification of organic pollutants at pollution incidents. Methods were developed and evaluated for both water and soil matrices.

The developed methods were easy to use and reliable, with detection limits suitable for emergency response application. Matrix interference was found to have some impact, but only when target compounds were present at or close to the MDL. Given that pollutants are generally present at concentrations above the MDL at emergency response incidents, matrix interference is unlikely to be a significant issue in real world applications.

The methods can analyse the same range of target compounds as is commonly screened for by regulatory environmental laboratories, whilst also being able to conduct library searches to tentatively identify non-target organic compounds. This provides the field officer with capabilities not otherwise available to them to gain a better understanding of the organic pollutants present at the scene of an incident. In addition, because the in-field method strongly relates to the laboratory method, the result obtained in the field can be used by the laboratory to triage the samples and to guide their analysis pathway.

Nevertheless, some compromises are necessary when conducting rapid in-field analyses. These compromises are mostly related to the reduced time available for the generation of results. The method for extracting samples is not as efficient as a laboratory-based sample extraction process because of the need to expedite the extraction process. The reduced analysis times on the portable GC–MS in comparison to its laboratory-based counterpart result in less selective separations, with the possibility that fewer compounds are being detected and identified by the portable GC–MS in comparison to a laboratory-based instrument. In addition, a portable GC–MS instrument requires a suitably trained operator to conduct the in-field analysis. It is for these reasons that the developed methods are highly suited to the endeavour of rapidly obtaining on-site intelligence, whereas laboratory-based analysis is critical for the confirmatory analysis required to support subsequent regulatory actions in relation to a pollution incident.

## Supplementary Information

Below is the link to the electronic supplementary material.

Supplementary file1 (PDF 670 KB)

## Data Availability

Not applicable.
