# Supplementary material for: Portable gas chromatography–mass spectrometry method for the in-field screening of organic pollutants in soil and water at pollution incidents
Source: Environ Sci Pollut Res Int. 2023 Jul 27;30(40):93088–102. doi: 10.1007/s11356-023-28648-w (PMC10447289; doi:10.1007/s11356-023-28648-w)
Supplement: Supplementary file 1 — Supplementary file1 (PDF 670 KB) [file 11356_2023_28648_MOESM1_ESM.pdf]

The tables of the supplementary information only provide the target compounds that could be detected. The internal standards and heavy compounds (PCBs and some heavy PAHs) that were not detectable on the portable GC-MS have not been included.

Table S1. Five repeat analyses on the portable GC-MS of the 5-ppm organic standard. A ✓ indicates that the compound was detected by the portable GC-MS in that analysis run, whereas a ✗ indicates that the compound was not detected within that analysis run. These results were used for determining the instrument detection limit (IDL).

5ppm Standard						
**Compound**	**Conc. (ppm)**	**1**	**2**	**3**	**4**	**5**
Phenol	20	✓	✓	✓	✓	✓
2-Chlorophenol	20	✓	✓	✓	✓	✓
1,4-Dichlorobenzene	5.0	✓	✓	✓	✓	✓
1,2-Dichlorobenzene	5.0	✓	✓	✓	✓	✓
2-Methylphenol (o-cresol)	20.0	✓	✓	✓	✓	✓
3+4-Methylphenol (m+p-cresol)	40.0	✓	✓	✓	✓	✓
Nitrobenzene	5.0	✓	✓	✓	✓	✓
2-Nitrophenol	20.0	✓	✓	✓	✓	✓
2,4-Dimethylphenol	20.0	✓	✓	✓	✓	✓
2,4-Dichlorophenol	20.0	✓	✓	✓	✓	✓
1,2,4-Trichlorobenzene	5.0	✓	✓	✓	✓	✓
Naphthalene	5.0	✓	✓	✓	✓	✓
2,6-dichlorophenol	20.0	✓	✓	✓	✓	✓
4-Chloro-3-Methylphenol	20.0	✓	✓	✓	✓	✓
1,2,4,5-Tetrachlorobenzene	5.0	✓	✓	✓	✓	✓
2,4,6-Trichlorophenol	20.0	✓	✓	✓	✓	✓
2,4,5-Trichlorophenol	20.0	✓	✓	✓	✓	✓
1,2,3,4-Tetrachlorobenzene	5.0	✓	✓	✓	✓	✓
Acenaphthylene	5.0	✓	✓	✓	✓	✓
Acenaphthene	5.0	✓	✓	✓	✓	✓
2,4-Dinitrophenol	20.0	✓	✓	✓	✓	✓
Pentachlorobenzene	5.0	✓	✓	✓	✓	✓
2,4-dinitrotoluene	5.1	✓	✓	✓	✓	✓
2,3,4,6-tetrachlorophenol	20	✓	✓	✓	✓	✓
2,3,5,6-Tetrachlorophenol	20.0	✓	✓	✓	✓	✓
Fluorene	5.0	✓	✓	✓	✓	✓
2-Methyl-4 6-Dinitrophenol	20.0	✓	✓	✓	✓	✓
Alpha-BHC	5.0	✓	✓	✓	✓	✓
Hexachlorobenzene	5.0	✓	✓	✓	✓	✓
Pentachlorophenol	20.0	✓	✓	✓	✓	✓
Beta or Gamma-BHC	5.0	✓	✓	✓	✓	✓
Pentachloronitrobenzene	5.0	✓	✓	✓	✓	✓
Phenanthrene	5.0	✓	✓	✓	✓	✓
Dinoseb	20.0	✓	✓	✓	✓	✓
Anthracene	5.0	✓	✓	✓	✓	✓
Delta-BHC	5.0	✓	✓	✓	✓	✓
Heptachlor	5.0	✓	✓	✓	✓	✓
Dibutyl phthalate	5.0	✓	✓	✓	✓	✓
Chlorpyrifos	5.0	✓	✓	✓	✓	✓
Aldrin	5.0	✓	✓	✓	✓	✓
Isodrin	5.0	✓	✓	✓	✓	✓
Heptachlor Epoxide	5.0	✓	✓	✓	✓	✓
Fluoranthene	5.0	✓	✓	✓	✓	✓
Gamma-Chlordane	5.0	✓	✓	✓	✓	✓
Alpha-Chlordane	5.0	✓	✓	✓	✓	✓
Endosulfan I	10.1	✓	✓	✓	✗	✗
Pyrene	5.0	✓	✓	✓	✓	✓
p,p’-DDE	5.0	✓	✓	✓	✓	✓
Dieldrin	5.0	✓	✓	✓	✓	✓
Endrin	5.0	✓	✓	✓	✓	✓
p,p’-DDD	5.0	✓	✓	✓	✓	✓
Endosulfan II	10.0	✓	✓	✓	✓	✓
Endrin Aldehyde	5.0	✓	✓	✓	✓	✓
p,p’-DDT	5.0	✓	✓	✓	✓	✓
Endosulfan Sulfate	10.0	✓	✓	✓	✓	✓
Bis(2-ethylhexyl) adipate	5.0	✓	✓	✓	✓	✓
Benzo (a) anthracene	5.0	✓	✓	✓	✓	✓
Chrysene	5.0	✓	✓	✓	✓	✓
Bis(2-ethylhexyl) phthalate	5.0	✓	✓	✓	✓	✓

Table S2. Seven repeat analyses on the portable GC-MS of the 0.5-ppm organic standard. A ✓ indicates that the compound was detected by the portable GC-MS in that analysis run, whereas a ✗ indicates that the compound was not detected within that analysis run. These results were used for determining the IDL.

0.5ppm Standard								
**Compound**	**Conc. (ppm)**	**1**	**2**	**3**	**4**	**5**	**6**	**7**
Phenol	2.0	✓	✓	✓	✓	✓	✓	✓
2-Chlorophenol	2.0	✓	✓	✓	✓	✓	✓	✗
1,4-Dichlorobenzene	0.5	✓	✓	✗	✗	✓	✓	✗
1,2-Dichlorobenzene	0.5	✓	✓	✗	✗	✗	✓	✗
2-Methylphenol (o-cresol)	2.0	✓	✓	✓	✓	✓	✓	✓
3+4-Methylphenol (m+p-cresol)	4.0	✓	✓	✓	✓	✓	✓	✓
Nitrobenzene	0.5	✓	✓	✓	✓	✓	✓	✓
2-Nitrophenol	2.0	✓	✓	✗	✗	✓	✓	✗
2,4-Dimethylphenol	2.0	✓	✓	✓	✓	✓	✓	✓
2,4-Dichlorophenol	2.0	✓	✓	✓	✓	✓	✓	✓
1,2,4-Trichlorobenzene	2.0	✓	✓	✗	✗	✗	✓	✓
Naphthalene	2.0	✓	✓	✓	✓	✓	✓	✓
2,6-dichlorophenol	2.0	✓	✓	✓	✓	✓	✓	✓
4-Chloro-3-Methylphenol	2.0	✓	✓	✓	✓	✓	✓	✓
1,2,4,5-Tetrachlorobenzene	2.0	✓	✓	✓	✓	✓	✓	✓
2,4,6-Trichlorophenol	2.0	✓	✓	✓	✓	✓	✓	✓
2,4,5-Trichlorophenol	2.0	✓	✓	✓	✓	✓	✓	✓
1,2,3,4-Tetrachlorobenzene	0.5	✓	✓	✓	✓	✓	✓	✓
Acenaphthylene	0.5	✓	✓	✓	✓	✓	✓	✓
Acenaphthene	0.5	✓	✓	✓	✓	✓	✓	✓
2,4-Dinitrophenol	2.0	✗	✓	✓	✓	✓	✓	✗
Pentachlorobenzene	0.5	✓	✓	✓	✓	✓	✓	✓
2,4-dinitrotoluene	0.5	✓	✓	✓	✓	✓	✓	✓
2,3,4,6-tetrachlorophenol	2.0	✓	✓	✓	✓	✓	✓	✓
2,3,5,6-Tetrachlorophenol	0.5	✓	✓	✓	✓	✓	✓	✓
Fluorene	0.5	✓	✓	✓	✓	✓	✓	✓
2-Methyl-4 6-Dinitrophenol	2.0	✓	✓	✓	✓	✓	✓	✓
Alpha-BHC	0.5	✓	✓	✓	✓	✓	✓	✓
Hexachlorobenzene	0.5	✓	✓	✓	✓	✓	✓	✓
Pentachlorophenol	2.0	✓	✓	✓	✓	✓	✓	✓
Beta or Gamma-BHC	0.5	✓	✓	✓	✓	✓	✓	✓
Pentachloronitrobenzene	0.5	✓	✓	✓	✓	✓	✓	✓
Phenanthrene	0.5	✓	✓	✓	✓	✓	✓	✓
Dinoseb	2.0	✓	✓	✓	✓	✓	✓	✓
Anthracene	0.5	✓	✓	✓	✓	✓	✓	✓
Delta-BHC	0.5	✓	✓	✓	✓	✓	✓	✓
Heptachlor	0.5	✓	✓	✓	✓	✓	✓	✓
Dibutyl phthalate	0.5	✓	✓	✓	✓	✓	✓	✓
Chlorpyrifos	0.5	✓	✓	✓	✓	✓	✓	✓
Aldrin	0.5	✓	✓	✓	✓	✓	✓	✓
Isodrin	0.5	✓	✓	✓	✓	✓	✓	✓
Heptachlor Epoxide	0.5	✓	✓	✓	✓	✓	✓	✓
Fluoranthene	0.5	✓	✓	✓	✓	✓	✓	✓
Gamma-Chlordane	0.5	✓	✓	✓	✓	✓	✓	✓
Alpha-Chlordane	0.5	✓	✓	✓	✓	✓	✓	✓
Endosulfan I	1.0	✓	✓	✓	✓	✓	✓	✓
Pyrene	0.5	✓	✓	✓	✓	✓	✓	✓
p,p'-DDE	0.5	✓	✓	✓	✓	✓	✓	✓
Dieldrin	0.5	✓	✓	✓	✓	✓	✓	✓
Endrin	0.5	✓	✓	✓	✓	✓	✓	✓
p,p'-DDD	0.5	✓	✓	✓	✓	✓	✓	✓
Endosulfan II	1.0	✓	✓	✓	✓	✓	✓	✓
Endrin Aldehyde	0.5	✓	✓	✓	✓	✓	✓	✓
p,p'-DDT	0.5	✓	✓	✓	✓	✓	✓	✓
Endosulfan Sulfate	1.0	✓	✓	✓	✓	✓	✓	✓
Bis(2-ethylhexyl) adipate	0.5	✓	✓	✓	✓	✓	✓	✓
Benzo (a) anthracene	0.5	✓	✓	✓	✓	✓	✓	✓
Chrysene	0.5	✓	✓	✓	✓	✓	✓	✓
Bis(2-ethylhexyl) phthalate	0.5	✓	✓	✓	✓	✓	✓	✓

Table S3. Seven repeat analyses on the portable GC-MS of the 0.15-ppm organic standard. A ✓ indicates that the compound was detected by the portable GC-MS in that analysis run, whereas a ✗ indicates that the compound was not detected within that analysis run. These results were obtained by loading the CME 6 times instead of 3 times. These results were used for determining the IDL.

**0.15 ppm Standard**								
**Compound**	**Conc. (ppm)**	**1**	**2**	**3**	**4**	**5**	**6**	**7**
Phenol	0.60	✓	✓	✓	✓	✓	✓	✓
2-Chlorophenol	0.60	✓	✗	✓	✓	✗	✗	✓
1,4-Dichlorobenzene	0.15	✓	✗	✗	✗	✗	✗	✗
1,2-Dichlorobenzene	0.15	✗	✗	✗	✗	✗	✗	✗
2-Methylphenol (o-cresol)	0.60	✓	✓	✓	✓	✓	✓	✓
3+4-Methylphenol (m+p-cresol)	1.2	✓	✓	✓	✓	✓	✓	✓
Nitrobenzene	0.15	✗	✗	✗	✗	✗	✗	✗
2-Nitrophenol	0.60	✓	✗	✓	✗	✗	✓	✓
2,4-Dimethylphenol	0.60	✓	✓	✓	✓	✓	✗	✓
2,4-Dichlorophenol	0.60	✓	✗	✓	✓	✓	✗	✓
1,2,4-Trichlorobenzene	0.15	✓	✗	✗	✗	✗	✗	✗
Naphthalene	0.15	✓	✓	✓	✗	✓	✓	✓
2,6-dichlorophenol	0.60	✓	✗	✓	✓	✓	✗	✓
4-Chloro-3-Methylphenol	0.60	✓	✓	✓	✓	✓	✗	✓
1,2,4,5-Tetrachlorobenzene	0.15	✓	✓	✓	✓	✓	✗	✓
2,4,6-Trichlorophenol	0.60	✓	✓	✓	✓	✓	✓	✓
2,4,5-Trichlorophenol	0.60	✓	✓	✓	✓	✓	✓	✓
1,2,3,4-Tetrachlorobenzene	0.15	✓	✗	✓	✓	✓	✗	✓
Acenaphthylene	0.15	✓	✓	✓	✓	✓	✓	✓
Acenaphthene	0.15	✓	✓	✓	✓	✓	✗	✓
2,4-Dinitrophenol	0.60	✗	✓	✗	✗	✗	✓	✗
Pentachlorobenzene	0.15	✓	✗	✓	✓	✓	✗	✓
2,4-dinitrotoluene	0.15	✓	✓	✓	✓	✓	✓	✓
2,3,4,6-tetrachlorophenol	0.60	✓	✓	✓	✓	✓	✓	✓
2,3,5,6-Tetrachlorophenol	0.60	✓	✗	✓	✓	✓	✗	✓
Fluorene	0.15	✓	✓	✓	✓	✓	✓	✓
2-Methyl-4 6-Dinitrophenol	0.60	✓	✓	✓	✓	✓	✓	✓
Alpha-BHC	0.15	✓	✓	✓	✓	✓	✓	✓
Hexachlorobenzene	0.15	✓	✓	✓	✓	✓	✓	✓
Pentachlorophenol	0.60	✓	✓	✓	✓	✓	✓	✓
Beta or Gamma-BHC	0.15	✓	✓	✓	✓	✓	✓	✓
Pentachloronitrobenzene	0.15	✓	✓	✓	✓	✓	✗	✓
Phenanthrene	0.15	✓	✓	✓	✓	✓	✓	✓
Dinoseb	0.60	✓	✓	✗	✗	✓	✗	✗
Anthracene	0.15	✓	✓	✓	✓	✓	✓	✓
Delta-BHC	0.15	✓	✓	✓	✓	✓	✓	✓
Heptachlor	0.15	✓	✓	✓	✓	✓	✗	✓
Dibutyl phthalate	0.15	✓	✓	✓	✓	✓	✓	✓
Chlorpyrifos	0.15	✓	✓	✓	✗	✓	✓	✓
Aldrin	0.15	✓	✓	✓	✓	✓	✓	✓
Isodrin	0.15	✓	✓	✓	✓	✓	✓	✓
Heptachlor Epoxide	0.15	✓	✓	✓	✗	✓	✓	✓
Fluoranthene	0.15	✓	✓	✓	✓	✓	✓	✓
Gamma-Chlordane	0.15	✓	✓	✓	✗	✓	✗	✗
Alpha-Chlordane	0.15	✓	✓	✓	✓	✓	✓	✓
Endosulfan I	0.30	✗	✓	✗	✗	✗	✓	✓
Pyrene	0.15	✓	✓	✓	✓	✗	✓	✓
p,p'-DDE	0.15	✓	✓	✓	✓	✓	✓	✓
Dieldrin	0.15	✗	✗	✓	✓	✓	✓	✓
Endrin	0.15	✓	✓	✗	✗	✓	✓	✗
p,p'-DDD	0.15	✗	✗	✗	✗	✗	✗	✓
Endosulfan II	0.30	✗	✗	✓	✗	✓	✓	✗
Endrin Aldehyde	0.15	✗	✗	✗	✓	✓	✗	✗
p,p'-DDT	0.15	✗	✓	✗	✗	✗	✓	✗
Endosulfan Sulfate	0.30	✗	✓	✗	✓	✗	✓	✓
Bis(2-ethylhexyl) adipate	0.15	✗	✗	✗	✗	✗	✗	✗
Benzo (a) anthracene	0.15	✓	✓	✓	✓	✓	✓	✓
Chrysene	0.15	✗	✗	✓	✓	✗	✗	✓
Bis(2-ethylhexyl) phthalate	0.15	✗	✓	✗	✗	✓	✓	✓

Table S4. Seven replicate analyses of the blank sand spiked to a final theoretical concentration after extraction of 0.5 – 4 ppm (low spiked soil) and 1 – 8 ppm (high spiked soil) respectively. L indicates the results for the low spiked soil, whereas H indicates the results for the high spiked soil. A ✓ indicates that the compound was detected by the portable GC-MS in that analysis run, whereas a ✗ indicates that the compound was not detected within that analysis run. These results were used in determining the method detection limit (MDL).

	Blank Sand															
**Compound**	**Conc.**		**1**		**2**		**3**		**4**		**5**		**6**		**7**	
	**L**	**H**	**L**	**H**	**L**	**H**	**L**	**H**	**L**	**H**	**L**	**H**	**L**	**H**	**L**	**H**
Phenol	2	4	✓	✓	✓	✓	✓	✓	✓	✓	✓	✓	✓	✓	✓	✓
2-Chlorophenol	2	4	✓	✓	✓	✓	✓	✓	✓	✓	✓	✓	✓	✓	✓	✓
1,4-Dichlorobenzene	0.5	1	✗	✓	✗	✓	✗	✓	✗	✓	✗	✓	✗	✓	✗	✓
1,2-Dichlorobenzene	0.5	1	✗	✓	✗	✓	✗	✓	✗	✓	✗	✓	✗	✓	✗	✓
2-Methylphenol (o-cresol)	2	4	✓	✓	✓	✓	✓	✓	✓	✓	✓	✓	✓	✓	✓	✓
3+4-Methylphenol (m+p-cresol)	4	8	✓	✓	✓	✓	✓	✓	✓	✓	✓	✓	✓	✓	✓	✓
Nitrobenzene	0.5	2	✗	✓	✗	✓	✗	✓	✗	✓	✗	✓	✗	✓	✗	✓
2-Nitrophenol	2	4	✗	✓	✗	✓	✗	✓	✗	✓	✗	✓	✗	✓	✗	✓
2,4-Dimethylphenol	2	4	✓	✓	✓	✓	✓	✓	✓	✓	✓	✓	✓	✓	✓	✓
2,4-Dichlorophenol	2	4	✓	✓	✓	✓	✓	✓	✓	✓	✓	✓	✓	✓	✓	✓
1,2,4-Trichlorobenzene	0.5	1	✗	✓	✗	✓	✗	✓	✗	✓	✗	✓	✗	✓	✗	✓
Naphthalene	0.5	1	✗	✓	✗	✓	✗	✓	✗	✓	✗	✓	✗	✓	✗	✓
2,6-dichlorophenol	2	4	✓	✓	✓	✓	✓	✓	✓	✓	✓	✓	✓	✓	✓	✓
4-Chloro-3-Methylphenol	2	4	✓	✓	✓	✓	✓	✓	✓	✓	✓	✓	✓	✓	✓	✓
1,2,4,5-Tetrachlorobenzene	0.5	2	✓	✓	✓	✓	✓	✓	✓	✓	✓	✓	✓	✓	✓	✓
2,4,6-Trichlorophenol	2	4	✓	✓	✓	✓	✓	✓	✓	✓	✓	✓	✓	✓	✓	✓
2,4,5-Trichlorophenol	2	4	✓	✓	✓	✓	✓	✓	✓	✓	✓	✓	✓	✓	✓	✓
1,2,3,4-Tetrachlorobenzene	0.5	1	✓	✓	✓	✓	✓	✓	✓	✓	✓	✓	✓	✓	✓	✓
Acenaphthylene	0.5	1	✓	✓	✓	✓	✓	✓	✓	✓	✓	✓	✓	✓	✓	✓
Acenaphthene	0.5	1	✓	✓	✓	✓	✓	✓	✓	✓	✓	✓	✓	✓	✓	✓
2,4-Dinitrophenol	2	4	✗	✗	✗	✗	✗	✗	✗	✗	✗	✗	✗	✗	✗	✗
Pentachlorobenzene	0.5	1	✓	✓	✓	✓	✓	✓	✓	✓	✓	✓	✓	✓	✓	✓
2,4-dinitrotoluene	0.5	1	✓	✓	✓	✓	✓	✓	✓	✓	✓	✓	✓	✓	✓	✓
2,3,4,6-tetrachlorophenol	2	4	✓	✓	✓	✓	✓	✓	✓	✓	✓	✓	✓	✓	✓	✓
2,3,5,6-Tetrachlorophenol	2	4	✓	✓	✓	✓	✓	✓	✓	✓	✓	✓	✓	✓	✓	✓
Fluorene	0.5	1	✓	✓	✓	✓	✓	✓	✓	✓	✓	✓	✓	✓	✓	✓
2-Methyl-4 6-Dinitrophenol	2	4	✗	✗	✗	✗	✗	✗	✗	✗	✗	✗	✗	✗	✗	✗
Alpha-BHC	0.5	1	✓	✓	✓	✓	✓	✓	✓	✓	✓	✓	✓	✓	✓	✓
Hexachlorobenzene	0.5	1	✓	✓	✓	✓	✓	✓	✓	✓	✓	✓	✓	✓	✓	✓
Pentachlorophenol	2	4	✓	✓	✓	✓	✓	✓	✓	✓	✓	✓	✓	✓	✓	✓
Beta or Gamma-BHC	0.5	1	✓	✓	✓	✓	✓	✓	✓	✓	✓	✓	✓	✓	✓	✓
Pentachloronitrobenzene	0.5	1	✓	✓	✓	✓	✓	✓	✓	✓	✓	✓	✓	✓	✓	✓
Phenanthrene	0.5	1	✓	✓	✓	✓	✓	✓	✓	✓	✓	✓	✓	✓	✓	✓
Dinoseb	2	4	✓	✓	✓	✓	✓	✓	✓	✓	✓	✓	✓	✓	✓	✓
Anthracene	0.5	1	✓	✓	✓	✓	✓	✓	✓	✓	✓	✓	✓	✓	✓	✓
Delta-BHC	0.5	1	✓	✓	✓	✓	✓	✓	✓	✓	✓	✓	✓	✓	✓	✓
Heptachlor	0.5	1	✓	✓	✓	✓	✓	✓	✓	✓	✓	✓	✓	✓	✓	✓
Dibutyl phthalate	0.5	1	✓	✓	✓	✓	✓	✓	✓	✓	✓	✓	✓	✓	✓	✓
Chlorpyrifos	0.5	1	✓	✓	✓	✓	✓	✓	✓	✓	✓	✓	✓	✓	✓	✓
Aldrin	0.5	1	✓	✓	✓	✓	✓	✓	✓	✓	✓	✓	✓	✓	✓	✓
Isodrin	0.5	1	✓	✓	✓	✓	✓	✓	✓	✓	✓	✓	✓	✓	✓	✓
Heptachlor Epoxide	0.5	1	✗	✓	✗	✓	✗	✓	✗	✓	✗	✓	✗	✓	✗	✓
Fluoranthene	0.5	1	✓	✓	✓	✓	✓	✓	✓	✓	✓	✓	✓	✓	✓	✓
Gamma-Chlordane	0.5	1	✗	✓	✗	✗	✗	✓	✗	✓	✗	✓	✗	✓	✗	✓
Alpha-Chlordane	0.5	1	✗	✓	✗	✗	✗	✓	✗	✓	✗	✓	✗	✓	✗	✓
Endosulfan I	1	2	✗	✓	✗	✓	✗	✓	✗	✓	✗	✓	✗	✓	✗	✓
Pyrene	0.5	1	✓	✓	✓	✓	✓	✓	✓	✓	✓	✓	✓	✓	✓	✓
p,p'-DDE	0.5	1	✓	✓	✓	✓	✓	✓	✓	✓	✓	✓	✓	✓	✓	✓
Dieldrin	0.5	1	✗	✗	✗	✗	✗	✓	✗	✓	✗	✓	✗	✓	✗	✓
Endrin	0.5	1	✗	✓	✗	✗	✗	✗	✗	✓	✗	✓	✗	✗	✗	✓
p,p'-DDD	0.5	1	✓	✓	✓	✓	✓	✓	✓	✓	✓	✓	✓	✓	✓	✓
Endosulfan II	1	2	✗	✓	✗	✗	✗	✓	✗	✓	✗	✓	✗	✓	✗	✓
Endrin Aldehyde	0.5	1	✗	✗	✗	✗	✗	✗	✗	✗	✗	✗	✗	✗	✗	✗
p,p'-DDT	0.5	1	✓	✓	✓	✓	✓	✓	✓	✓	✓	✓	✓	✓	✓	✓
Endosulfan Sulfate	1	2	✗	✗	✗	✗	✗	✗	✗	✗	✗	✓	✗	✓	✗	✗
Bis(2-ethylhexyl) adipate	0.5	1	✗	✓	✗	✓	✗	✓	✗	✓	✗	✓	✗	✓	✗	✓
Benzo (a) anthracene	0.5	1	✓	✓	✓	✓	✓	✓	✓	✓	✓	✓	✓	✓	✓	✓
Chrysene	0.5	1	✓	✓	✓	✓	✓	✓	✓	✓	✓	✓	✓	✓	✓	✓
Bis(2-ethylhexyl) phthalate	0.5	1	✗	✓	✗	✓	✗	✓	✗	✓	✗	✓	✗	✓	✗	✓

Table S5. Seven replicate analyses of the Soil 1 spiked to a final theoretical concentration after extraction of 0.5 – 4 ppm (low spiked soil) and 1 – 8 ppm (high spiked soil) respectively. L indicates the results for the low spiked soil, whereas H indicates the results for the high spiked soil. A ✓ indicates that the compound was detected by the portable GC-MS in that analysis run, whereas a ✗ indicates that the compound was not detected within that analysis run. These results were used in determining the MDL.

Soil 1																
**Compound**	**Conc.**		**1**		**2**		**3**		**4**		**5**		**6**		**7**	
	**L**	**H**	**L**	**H**	**L**	**H**	**L**	**H**	**L**	**H**	**L**	**H**	**L**	**H**	**L**	**H**
Phenol	2	4	✓	✓	✓	✓	✓	✓	✓	✓	✓	✓	✓	✓	✓	✓
2-Chlorophenol	2	4	✓	✓	✓	✓	✓	✓	✓	✓	✓	✓	✓	✓	✓	✓
1,4-Dichlorobenzene	0.5	1	✗	✓	✗	✓	✗	✓	✗	✓	✗	✓	✗	✓	✗	✓
1,2-Dichlorobenzene	0.5	1	✗	✓	✗	✓	✗	✓	✗	✓	✗	✓	✗	✓	✗	✓
2-Methylphenol (o-cresol)	2	4	✓	✓	✓	✓	✓	✓	✓	✓	✓	✓	✓	✓	✓	✓
3+4-Methylphenol (m+p-cresol)	4	8	✓	✓	✓	✓	✓	✓	✓	✓	✓	✓	✓	✓	✓	✓
Nitrobenzene	0.5	2	✗	✓	✗	✓	✗	✓	✗	✓	✗	✓	✗	✓	✗	✓
2-Nitrophenol	2	4	✗	✓	✗	✓	✗	✓	✗	✓	✗	✓	✗	✓	✗	✓
2,4-Dimethylphenol	2	4	✓	✓	✓	✓	✓	✓	✓	✓	✓	✓	✓	✓	✓	✓
2,4-Dichlorophenol	2	4	✓	✓	✓	✓	✓	✓	✓	✓	✓	✓	✓	✓	✓	✓
1,2,4-Trichlorobenzene	0.5	1	✗	✓	✗	✓	✗	✓	✗	✓	✗	✓	✗	✓	✗	✓
Naphthalene	0.5	1	✓	✓	✓	✓	✓	✓	✓	✓	✓	✓	✓	✓	✓	✓
2,6-dichlorophenol	2	4	✓	✓	✓	✓	✓	✓	✓	✓	✓	✓	✓	✓	✓	✓
4-Chloro-3-Methylphenol	2	4	✓	✓	✓	✓	✓	✓	✓	✓	✓	✓	✓	✓	✓	✓
1,2,4,5-Tetrachlorobenzene	0.5	2	✓	✓	✓	✓	✓	✓	✓	✓	✓	✓	✓	✓	✓	✓
2,4,6-Trichlorophenol	2	4	✓	✓	✓	✓	✓	✓	✓	✓	✓	✓	✓	✓	✓	✓
2,4,5-Trichlorophenol	2	4	✓	✓	✓	✓	✓	✓	✓	✓	✓	✓	✓	✓	✓	✓
1,2,3,4-Tetrachlorobenzene	0.5	1	✓	✓	✓	✓	✓	✓	✓	✓	✓	✓	✓	✓	✓	✓
Acenaphthylene	0.5	1	✓	✓	✓	✓	✓	✓	✓	✓	✓	✓	✓	✓	✓	✓
Acenaphthene	0.5	1	✓	✓	✓	✓	✓	✓	✓	✓	✓	✓	✓	✓	✓	✓
2,4-Dinitrophenol	2	4	✗	✗	✗	✗	✗	✗	✗	✗	✗	✗	✗	✗	✗	✗
Pentachlorobenzene	0.5	1	✓	✓	✓	✓	✓	✓	✓	✓	✓	✓	✓	✓	✓	✓
2,4-dinitrotoluene	0.5	1	✓	✓	✓	✓	✓	✓	✓	✓	✓	✓	✓	✓	✓	✓
2,3,4,6-tetrachlorophenol	2	4	✓	✓	✓	✓	✓	✓	✓	✓	✓	✓	✓	✓	✓	✓
2,3,5,6-Tetrachlorophenol	2	4	✓	✓	✓	✓	✓	✓	✓	✓	✓	✓	✓	✓	✓	✓
Fluorene	0.5	1	✓	✓	✓	✓	✓	✓	✓	✓	✓	✓	✓	✓	✓	✓
2-Methyl-4 6-Dinitrophenol	2	4	✗	✓	✗	✓	✗	✓	✗	✓	✗	✓	✗	✓	✗	✓
Alpha-BHC	0.5	1	✓	✓	✓	✓	✓	✓	✓	✓	✓	✓	✓	✓	✓	✓
Hexachlorobenzene	0.5	1	✓	✓	✓	✓	✓	✓	✓	✓	✓	✓	✓	✓	✓	✓
Pentachlorophenol	2	4	✓	✓	✓	✓	✓	✓	✓	✓	✓	✓	✓	✓	✓	✓
Beta or Gamma-BHC	0.5	1	✓	✓	✓	✓	✓	✓	✓	✓	✓	✓	✓	✓	✓	✓
Pentachloronitrobenzene	0.5	1	✓	✓	✓	✓	✓	✓	✓	✓	✓	✓	✓	✓	✓	✓
Phenanthrene	0.5	1	✓	✓	✓	✓	✓	✓	✓	✓	✓	✓	✓	✓	✓	✓
Dinoseb	2	4	✓	✓	✓	✓	✓	✓	✓	✓	✓	✓	✓	✓	✓	✓
Anthracene	0.5	1	✓	✓	✓	✓	✓	✓	✓	✓	✓	✓	✓	✓	✓	✓
Delta-BHC	0.5	1	✓	✓	✓	✓	✓	✓	✓	✓	✓	✓	✓	✓	✓	✓
Heptachlor	0.5	1	✓	✓	✓	✓	✓	✓	✓	✓	✓	✓	✓	✓	✓	✓
Dibutyl phthalate	0.5	1	✓	✓	✓	✓	✓	✓	✓	✓	✓	✓	✓	✓	✓	✓
Chlorpyrifos	0.5	1	✓	✓	✓	✓	✓	✓	✓	✓	✓	✓	✓	✓	✓	✓
Aldrin	0.5	1	✓	✓	✓	✓	✓	✓	✓	✓	✓	✓	✓	✓	✓	✓
Isodrin	0.5	1	✓	✓	✓	✓	✓	✓	✓	✓	✓	✓	✓	✓	✓	✓
Heptachlor Epoxide	0.5	1	✗	✓	✗	✓	✗	✓	✗	✓	✗	✓	✗	✓	✗	✓
Fluoranthene	0.5	1	✓	✓	✓	✓	✓	✓	✓	✓	✓	✓	✓	✓	✓	✓
Gamma-Chlordane	0.5	1	✗	✓	✗	✓	✗	✓	✗	✓	✗	✓	✗	✓	✗	✓
Alpha-Chlordane	0.5	1	✗	✓	✗	✓	✗	✓	✗	✓	✗	✓	✗	✓	✗	✓
Endosulfan I	1	2	✗	✓	✗	✓	✗	✓	✗	✓	✗	✓	✗	✓	✗	✓
Pyrene	0.5	1	✓	✓	✓	✓	✓	✓	✓	✓	✓	✓	✓	✓	✓	✓
p,p’-DDE	0.5	1	✓	✓	✓	✓	✓	✓	✓	✓	✓	✓	✓	✓	✓	✓
Dieldrin	0.5	1	✗	✓	✗	✓	✗	✓	✗	✓	✗	✓	✗	✓	✗	✓
Endrin	0.5	1	✗	✗	✗	✗	✗	✗	✗	✗	✗	✗	✗	✗	✗	✗
p,p’-DDD	0.5	1	✓	✓	✓	✓	✓	✓	✓	✓	✓	✓	✓	✓	✓	✓
Endosulfan II	1	2	✗	✓	✗	✓	✗	✓	✗	✓	✗	✓	✗	✓	✗	✓
Endrin Aldehyde	0.5	1	✗	✗	✗	✗	✗	✗	✗	✗	✗	✗	✗	✗	✗	✗
p,p’-DDT	0.5	1	✗	✓	✗	✓	✗	✓	✗	✓	✗	✓	✗	✓	✗	✓
Endosulfan Sulfate	1	2	✗	✓	✗	✓	✗	✓	✗	✗	✗	✓	✗	✓	✗	✓
Bis(2-ethylhexyl) adipate	0.5	1	✗	✓	✗	✓	✗	✓	✗	✓	✗	✓	✗	✓	✗	✓
Benzo (a) anthracene	0.5	1	✓	✓	✓	✓	✓	✓	✓	✓	✓	✓	✓	✓	✓	✓
Chrysene	0.5	1	✓	✓	✓	✓	✓	✓	✓	✓	✓	✓	✓	✓	✓	✓
Bis(2-ethylhexyl) phthalate	0.5	1	✓	✓	✓	✓	✓	✓	✓	✓	✓	✓	✓	✓	✓	✓

Table S6. Seven replicate analyses of the Soil 2 spiked to a final theoretical concentration after extraction of 0.5 – 4 ppm (low spiked soil) and 1 – 8 ppm (high spiked soil) respectively. L indicates the results for the low spiked soil, whereas H indicates the results for the high spiked soil. A ✓ indicates that the compound was detected by the portable GC-MS in that analysis run, whereas a × indicates that the compound was not detected within that analysis run. These results were used in determining the MDL.

Soil 2																
Compound	Conc.		1		2		3		4		5		6		7	
	L	H	L	H	L	H	L	H	L	H	L	H	L	H	L	H
Phenol	2	4	✓	✓	✓	✓	✓	✓	✓	✓	✓	✓	✓	✓	✓	✓
2-Chlorophenol	2	4	✓	✓	✓	✓	✓	✓	✓	✓	✓	✓	✓	✓	✓	✓
1,4-Dichlorobenzene	0.5	1	×	✓	×	✓	×	✓	×	✓	×	✓	×	✓	×	✓
1,2-Dichlorobenzene	0.5	1	×	✓	×	✓	×	✓	×	✓	×	✓	×	✓	×	✓
2-Methylphenol (o-cresol)	2	4	✓	✓	✓	✓	✓	✓	✓	✓	✓	✓	✓	✓	✓	✓
3+4-Methylphenol (m+p-cresol)	4	8	✓	✓	✓	✓	✓	✓	✓	✓	✓	✓	✓	✓	✓	✓
Nitrobenzene	0.5	2	×	✓	×	✓	×	✓	×	✓	×	✓	×	✓	×	✓
2-Nitrophenol	2	4	✓	✓	✓	✓	✓	✓	✓	✓	✓	✓	✓	✓	✓	✓
2,4-Dimethylphenol	2	4	✓	✓	✓	✓	✓	✓	✓	✓	✓	✓	✓	✓	✓	✓
2,4-Dichlorophenol	2	4	✓	✓	✓	✓	✓	✓	✓	✓	✓	✓	✓	✓	✓	✓
1,2,4-Trichlorobenzene	0.5	1	×	✓	×	✓	×	✓	×	✓	×	✓	×	✓	×	✓
Naphthalene	0.5	1	✓	✓	✓	✓	✓	✓	✓	✓	✓	✓	✓	✓	✓	✓
2,6-dichlorophenol	2	4	✓	✓	✓	✓	✓	✓	✓	✓	✓	✓	✓	✓	✓	✓
4-Chloro-3-Methylphenol	2	4	✓	✓	✓	✓	✓	✓	✓	✓	✓	✓	✓	✓	✓	✓
1,2,4,5-Tetrachlorobenzene	0.5	2	✓	✓	✓	✓	✓	✓	✓	✓	✓	✓	✓	✓	✓	✓
2,4,6-Trichlorophenol	2	4	✓	✓	✓	✓	✓	✓	✓	✓	✓	✓	✓	✓	✓	✓
2,4,5-Trichlorophenol	2	4	✓	✓	✓	✓	✓	✓	✓	✓	✓	✓	✓	✓	✓	✓
1,2,3,4-Tetrachlorobenzene	0.5	1	✓	✓	✓	✓	✓	✓	✓	✓	✓	✓	✓	✓	✓	✓
Acenaphthylene	0.5	1	✓	✓	✓	✓	✓	✓	✓	✓	✓	✓	✓	✓	✓	✓
Acenaphthene	0.5	1	✓	✓	✓	✓	✓	✓	✓	✓	✓	✓	✓	✓	✓	✓
2,4-Dinitrophenol	2	4	×	×	×	×	×	×	×	×	×	×	×	×	×	×
Pentachlorobenzene	0.5	1	✓	✓	✓	✓	✓	✓	✓	✓	✓	✓	✓	✓	✓	✓
2,4-dinitrotoluene	0.5	1	×	✓	×	✓	×	✓	×	✓	×	✓	×	×	×	×
2,3,4,6-tetrachlorophenol	2	4	✓	✓	✓	✓	✓	✓	✓	✓	✓	✓	✓	✓	✓	✓
2,3,5,6-Tetrachlorophenol	2	4	✓	✓	✓	✓	✓	✓	✓	✓	✓	✓	✓	✓	✓	✓
Fluorene	0.5	1	✓	✓	✓	✓	✓	✓	✓	✓	✓	✓	✓	✓	✓	✓
2-Methyl-4 6-Dinitrophenol	2	4	✓	✓	✓	✓	✓	✓	✓	✓	✓	✓	✓	✓	✓	✓
Alpha-BHC	0.5	1	✓	✓	✓	✓	✓	✓	✓	✓	✓	✓	✓	✓	✓	✓
Hexachlorobenzene	0.5	1	✓	✓	✓	✓	✓	✓	✓	✓	✓	✓	✓	✓	✓	✓
Pentachlorophenol	2	4	✓	✓	✓	✓	✓	✓	✓	✓	✓	✓	✓	✓	✓	✓
Beta or Gamma-BHC	0.5	1	✓	✓	✓	✓	✓	✓	✓	✓	✓	✓	✓	✓	✓	✓
Pentachloronitrobenzene	0.5	1	✓	✓	✓	✓	✓	✓	✓	✓	✓	✓	✓	✓	✓	✓
Phenanthrene	0.5	1	✓	✓	✓	✓	✓	✓	✓	✓	✓	✓	✓	✓	✓	✓
Dinoseb	2	4	✓	✓	✓	✓	✓	✓	✓	✓	✓	✓	✓	✓	✓	✓
Anthracene	0.5	1	✓	✓	✓	✓	✓	✓	✓	✓	✓	✓	✓	✓	✓	✓
Delta-BHC	0.5	1	✓	✓	✓	✓	✓	✓	✓	✓	✓	✓	✓	✓	✓	✓
Heptachlor	0.5	1	✓	✓	✓	✓	✓	✓	✓	✓	✓	✓	✓	✓	✓	✓
Dibutyl phthalate	0.5	1	✓	✓	✓	✓	✓	✓	✓	✓	✓	✓	✓	✓	✓	✓
Chlorpyrifos	0.5	1	×	✓	×	✓	×	✓	×	✓	×	✓	×	✓	×	×
Aldrin	0.5	1	×	✓	×	✓	×	✓	×	✓	×	✓	×	✓	×	✓
Isodrin	0.5	1	✓	✓	✓	✓	✓	✓	✓	✓	✓	✓	✓	✓	✓	✓
Heptachlor Epoxide	0.5	1	×	✓	×	×	×	✓	×	✓	×	✓	×	✓	×	✓
Fluoranthene	0.5	1	✓	✓	✓	✓	✓	✓	✓	✓	✓	✓	✓	✓	✓	✓
Gamma-Chlordane	0.5	1	×	✓	×	✓	×	✓	×	✓	×	✓	×	✓	×	✓
Alpha-Chlordane	0.5	1	×	✓	×	✓	×	✓	×	×	×	✓	×	✓	×	✓
Endosulfan I	1	2	×	✓	×	✓	×	✓	×	✓	×	✓	×	✓	×	✓
Pyrene	0.5	1	✓	✓	✓	✓	✓	✓	✓	✓	✓	✓	✓	✓	✓	✓
p,p’-DDE	0.5	1	✓	✓	✓	✓	✓	✓	✓	✓	✓	✓	✓	✓	✓	✓
Dieldrin	0.5	1	×	✓	×	✓	×	✓	×	✓	×	✓	×	✓	×	✓
Endrin	0.5	1	×	×	×	×	×	×	×	×	×	×	×	×	×	×
p,p’-DDD	0.5	1	✓	✓	✓	✓	✓	✓	✓	✓	✓	✓	✓	✓	✓	✓
Endosulfan II	1	2	×	✓	×	×	×	✓	×	✓	×	×	×	✓	×	✓
Endrin Aldehyde	0.5	1	×	×	×	×	×	×	×	×	×	×	×	×	×	×
p,p’-DDT	0.5	1	×	✓	×	✓	×	✓	×	✓	×	✓	×	✓	×	✓
Endosulfan Sulfate	1	2	×	✓	×	×	×	✓	×	✓	×	✓	×	✓	×	✓
Bis(2-ethylhexyl) adipate	0.5	1	×	✓	×	✓	×	✓	×	✓	×	✓	×	✓	×	✓
Benzo (a) anthracene	0.5	1	✓	✓	✓	✓	✓	✓	✓	✓	✓	✓	✓	✓	✓	✓
Chrysene	0.5	1	✓	✓	✓	✓	✓	✓	✓	✓	✓	✓	✓	✓	✓	✓
Bis(2-ethylhexyl) phthalate	0.5	1	×	✓	×	✓	×	✓	×	✓	×	✓	×	✓	×	✓

Table S7. Replicate analyses for 7 out of 14 different river water matrices spiked to a final theoretical concentration after extraction of 0.2 – 1.6 ppm (low spiked water). Included in the table are also the observations for a single river water sample that was spiked to a final theoretical concentration after extraction of 0.4 – 3.2 ppm (high spiked water) and analysed as seven repeats. L = low spiked water, whereas H = high spiked water. ✓ indicates that the compound was detected by the portable GC-MS in that analysis run, while × indicates that the compound was not detected within that analysis run. These results were used in determining the MDL.

Water																
Compound	Conc.		1		2		3		4		5		6		7	
	L	H	L	H	L	H	L	H	L	H	L	H	L	H	L	H
Phenol	0.8	1.6	✓	✓	✓	✓	✓	✓	✓	✓	✓	✓	✓	✓	✓	✓
2-Chlorophenol	0.8	1.6	✓	✓	✓	✓	✓	✓	✓	✓	✓	✓	✓	✓	✓	✓
1,4-Dichlorobenzene	0.2	0.4	✓	✓	✓	✓	✓	✓	✓	✓	✓	✓	✓	✓	✓	✓
1,2-Dichlorobenzene	0.2	0.4	✓	✓	✓	✓	✓	✓	✓	✓	✓	✓	✓	✓	✓	✓
2-Methylphenol (o-cresol)	0.8	1.6	✓	✓	✓	✓	✓	✓	✓	✓	✓	✓	✓	✓	✓	✓
3+4-Methylphenol (m+p-cresol)	1.6	3.2	✓	✓	✓	✓	✓	✓	✓	✓	✓	✓	✓	✓	✓	✓
Nitrobenzene	0.2	0.4	✓	✓	✓	✓	✓	✓	✓	✓	✓	✓	✓	✓	✓	✓
2-Nitrophenol	0.8	1.6	✓	✓	✓	✓	✓	✓	✓	✓	✓	✓	✓	✓	✓	✓
2,4-Dimethylphenol	0.8	1.6	✓	✓	✓	✓	✓	✓	✓	✓	✓	✓	✓	✓	✓	✓
2,4-Dichlorophenol	0.8	1.6	✓	✓	✓	✓	✓	✓	✓	✓	✓	✓	✓	✓	✓	✓
1,2,4-Trichlorobenzene	0.2	0.4	✓	✓	✓	✓	✓	✓	✓	✓	✓	✓	✓	✓	✓	✓
Naphthalene	0.2	0.4	✓	✓	✓	✓	✓	✓	✓	✓	✓	✓	✓	✓	✓	✓
2,6-dichlorophenol	0.8	1.6	✓	✓	✓	✓	✓	✓	✓	✓	✓	✓	✓	✓	✓	✓
4-Chloro-3-Methylphenol	0.8	1.6	✓	✓	✓	✓	✓	✓	✓	✓	✓	✓	✓	✓	✓	✓
1,2,4,5-Tetrachlorobenzene	0.2	0.4	✓	✓	✓	✓	✓	✓	✓	✓	✓	✓	✓	✓	✓	✓
2,4,6-Trichlorophenol	0.8	1.6	✓	✓	✓	✓	✓	✓	✓	✓	✓	✓	✓	✓	✓	✓
2,4,5-Trichlorophenol	0.8	1.6	✓	✓	✓	✓	✓	✓	✓	✓	✓	✓	✓	✓	✓	✓
1,2,3,4-Tetrachlorobenzene	0.2	0.4	✓	✓	✓	✓	✓	✓	✓	✓	✓	✓	✓	✓	✓	✓
Acenaphthylene	0.2	0.4	✓	✓	✓	✓	✓	✓	✓	✓	✓	✓	✓	✓	✓	✓
Acenaphthene	0.2	0.4	✓	✓	✓	✓	✓	✓	✓	✓	✓	✓	✓	✓	✓	✓
2,4-Dinitrophenol	0.8	1.6	×	×	×	×	×	×	×	×	×	×	×	×	×	×
Pentachlorobenzene	0.2	0.4	✓	✓	✓	✓	✓	✓	✓	✓	✓	✓	✓	✓	✓	✓
2,4-dinitrotoluene	0.2	0.4	✓	✓	✓	✓	✓	✓	✓	✓	✓	✓	✓	✓	✓	✓
2,3,4,6-tetrachlorophenol	0.8	1.6	✓	✓	✓	✓	✓	✓	✓	✓	✓	✓	✓	✓	✓	✓
2,3,5,6-Tetrachlorophenol	0.8	1.6	✓	✓	✓	✓	✓	✓	✓	✓	✓	✓	✓	✓	✓	✓
Fluorene	0.2	0.4	✓	✓	✓	✓	✓	✓	✓	✓	✓	✓	✓	✓	✓	✓
2-Methyl-4 6-Dinitrophenol	0.8	1.6	×	×	×	×	×	×	×	×	×	×	×	×	×	×
Alpha-BHC	0.2	0.4	✓	✓	✓	✓	✓	✓	✓	✓	✓	✓	✓	✓	✓	✓
Hexachlorobenzene	0.2	0.4	✓	✓	✓	✓	✓	✓	✓	✓	✓	✓	✓	✓	✓	✓
Pentachlorophenol	0.8	1.6	✓	✓	✓	✓	✓	✓	✓	✓	✓	✓	✓	✓	✓	✓
Beta or Gamma-BHC	0.2	0.4	✓	✓	✓	✓	✓	✓	✓	✓	✓	✓	✓	✓	✓	✓
Pentachloronitrobenzene	0.2	0.4	✓	✓	✓	✓	✓	✓	✓	✓	✓	✓	✓	✓	✓	✓
Phenanthrene	0.2	0.4	✓	✓	✓	✓	✓	✓	✓	✓	✓	✓	✓	✓	✓	✓
Dinoseb	0.8	1.6	✓	✓	✓	✓	✓	✓	✓	✓	✓	✓	✓	✓	✓	✓
Anthracene	0.2	0.4	✓	✓	✓	✓	✓	✓	✓	✓	✓	✓	✓	✓	✓	✓
Delta-BHC	0.2	0.4	✓	✓	✓	✓	✓	✓	✓	✓	✓	✓	✓	✓	✓	✓
Heptachlor	0.2	0.4	✓	✓	✓	✓	✓	✓	✓	✓	✓	✓	✓	✓	✓	✓
Dibutyl phthalate	0.2	0.4	✓	✓	✓	✓	✓	✓	✓	✓	✓	✓	✓	✓	✓	✓
Chlorpyrifos	0.2	0.4	✓	✓	✓	✓	✓	✓	✓	✓	✓	✓	✓	✓	✓	✓
Aldrin	0.2	0.4	✓	✓	✓	✓	✓	✓	✓	✓	✓	✓	✓	✓	✓	✓
Isodrin	0.2	0.4	✓	✓	✓	✓	✓	✓	✓	✓	✓	✓	✓	✓	✓	✓
Heptachlor Epoxide	0.2	0.4	×	✓	×	✓	×	✓	×	✓	×	✓	×	✓	×	✓
Fluoranthene	0.2	0.4	✓	✓	✓	✓	✓	✓	✓	✓	✓	✓	✓	✓	✓	✓
Gamma-Chlordane	0.2	0.4	×	✓	×	✓	×	✓	×	✓	×	✓	×	✓	×	✓
Alpha-Chlordane	0.2	0.4	×	✓	×	✓	×	✓	×	✓	×	✓	×	✓	×	✓
Endosulfan I	0.4	0.8	×	✓	×	✓	×	✓	×	✓	×	✓	×	✓	×	✓
Pyrene	0.2	0.4	✓	✓	✓	✓	✓	✓	✓	✓	✓	✓	✓	✓	✓	✓
p,p'-DDE	0.2	0.4	✓	✓	✓	✓	✓	✓	✓	✓	✓	✓	✓	✓	✓	✓
Dieldrin	0.2	0.4	×	✓	×	✓	×	✓	×	✓	×	✓	×	✓	×	✓
Endrin	0.2	0.4	×	×	×	✓	×	✓	×	×	×	×	×	×	×	×
p,p'-DDD	0.2	0.4	✓	✓	✓	✓	✓	✓	✓	✓	✓	✓	✓	✓	✓	✓
Endosulfan II	0.4	0.8	×	✓	×	✓	×	✓	×	✓	×	✓	×	✓	×	✓
Endrin Aldehyde	0.2	0.4	×	×	×	×	×	×	×	×	×	×	×	×	×	×
p,p'-DDT	0.2	0.4	×	✓	×	✓	×	✓	×	✓	×	✓	×	✓	×	✓
Endosulfan Sulfate	0.4	0.8	×	✓	×	✓	×	✓	×	✓	×	✓	×	✓	×	✓
Bis(2-ethylhexyl) adipate	0.2	0.4	×	✓	×	✓	×	✓	×	✓	×	✓	×	✓	×	✓
Benzo (a) anthracene	0.2	0.4	✓	✓	✓	✓	✓	✓	✓	✓	✓	✓	✓	✓	✓	✓
Chrysene	0.2	0.4	✓	✓	✓	✓	✓	✓	✓	✓	✓	✓	✓	✓	✓	✓
Bis(2-ethylhexyl) phthalate	0.2	0.4	×	✓	×	✓	×	✓	×	✓	×	✓	×	✓	×	✓

Table S8. Replicate analysis results for the other 7 out of 14 different river water matrices spiked to a final theoretical concentration after extraction of 0.2 – 1.6 ppm (low spiked water). L indicates the results for the low spiked water. A ✓ indicates that the compound was detected by the portable GC-MS in that analysis run, whereas a ✗ indicates that the compound was not detected within that analysis run. These results were used in determining the MDL.

		River						
**Compound**	**Conc.**	**8**	**9**	**10**	**11**	**12**	**13**	**14**
	**L**	**L**	**L**	**L**	**L**	**L**	**L**	**L**
Phenol	0.8	✓	✗	✗	✗	✗	✗	✗
2-Chlorophenol	0.8	✓	✓	✓	✓	✓	✓	✓
1,4-Dichlorobenzene	0.2	✓	✓	✓	✓	✓	✓	✓
1,2-Dichlorobenzene	0.2	✓	✓	✓	✓	✓	✓	✓
2-Methylphenol (o-cresol)	0.8	✓	✓	✓	✓	✓	✓	✓
3+4-Methylphenol (m+p-cresol)	1.6	✓	✓	✓	✓	✓	✓	✓
Nitrobenzene	0.2	✓	✓	✓	✓	✓	✓	✓
2-Nitrophenol	0.8	✓	✓	✓	✓	✓	✓	✓
2,4-Dimethylphenol	0.8	✓	✓	✓	✓	✓	✓	✓
2,4-Dichlorophenol	0.8	✓	✓	✓	✓	✓	✓	✓
1,2,4-Trichlorobenzene	0.2	✓	✓	✓	✓	✓	✓	✓
Naphthalene	0.2	✓	✓	✓	✓	✓	✓	✓
2,6-dichlorophenol	0.8	✓	✓	✓	✓	✓	✓	✓
4-Chloro-3-Methylphenol	0.8	✓	✓	✓	✓	✓	✓	✓
1,2,4,5-Tetrachlorobenzene	0.2	✓	✓	✓	✓	✓	✓	✓
2,4,6-Trichlorophenol	0.8	✓	✓	✓	✓	✓	✓	✓
2,4,5-Trichlorophenol	0.8	✓	✓	✓	✓	✓	✓	✓
1,2,3,4-Tetrachlorobenzene	0.2	✓	✓	✓	✓	✓	✓	✓
Acenaphthylene	0.2	✓	✓	✓	✓	✓	✓	✓
Acenaphthene	0.2	✓	✓	✓	✓	✓	✓	✓
2,4-Dinitrophenol	0.8	✗	✗	✗	✗	✗	✗	✗
Pentachlorobenzene	0.2	✓	✓	✓	✓	✓	✓	✓
2,4-dinitrotoluene	0.2	✓	✓	✓	✓	✓	✓	✓
2,3,4,6-tetrachlorophenol	0.8	✓	✓	✓	✓	✓	✓	✓
2,3,5,6-Tetrachlorophenol	0.8	✓	✓	✓	✓	✓	✓	✓
Fluorene	0.2	✓	✓	✓	✓	✓	✓	✓
2-Methyl-4 6-Dinitrophenol	0.8	✗	✗	✗	✗	✓	✓	✓
Alpha-BHC	0.2	✓	✓	✓	✓	✓	✓	✓
Hexachlorobenzene	0.2	✓	✓	✓	✓	✓	✓	✓
Pentachlorophenol	0.8	✓	✓	✓	✓	✓	✓	✓
Beta or Gamma-BHC	0.2	✗	✓	✓	✓	✓	✓	✓
Pentachloronitrobenzene	0.2	✓	✓	✓	✓	✓	✓	✓
Phenanthrene	0.2	✓	✓	✓	✓	✓	✓	✓
Dinoseb	0.8	✓	✓	✓	✓	✓	✓	✓
Anthracene	0.2	✓	✓	✓	✓	✓	✓	✓
Delta-BHC	0.2	✓	✓	✓	✓	✓	✓	✓
Heptachlor	0.2	✓	✓	✓	✓	✓	✓	✓
Dibutyl phthalate	0.2	✓	✓	✓	✓	✓	✓	✓
Chlorpyrifos	0.2	✓	✓	✓	✓	✓	✓	✓
Aldrin	0.2	✓	✓	✓	✓	✓	✓	✓
Isodrin	0.2	✓	✓	✓	✓	✓	✓	✓
Heptachlor Epoxide	0.2	✗	✓	✓	✗	✓	✓	✓
Fluoranthene	0.2	✓	✓	✓	✓	✓	✓	✓
Gamma-Chlordane	0.2	✓	✓	✓	✓	✓	✓	✓
Alpha-Chlordane	0.2	✓	✓	✓	✓	✓	✓	✓
Endosulfan I	0.4	✗	✓	✓	✓	✗	✗	✓
Pyrene	0.2	✓	✓	✓	✓	✓	✓	✓
p,p'-DDE	0.2	✓	✓	✓	✓	✓	✓	✓
Dieldrin	0.2	✗	✗	✗	✗	✗	✗	✗
Endrin	0.2	✗	✗	✗	✗	✗	✗	✗
p,p'-DDD	0.2	✓	✓	✓	✓	✓	✓	✓
Endosulfan II	0.4	✗	✗	✗	✗	✗	✗	✓
Endrin Aldehyde	0.2	✗	✗	✗	✗	✗	✗	✗
p,p'-DDT	0.2	✗	✗	✗	✗	✗	✗	✗
Endosulfan Sulfate	0.4	✗	✗	✗	✗	✗	✗	✗
Bis(2-ethylhexyl) adipate	0.2	✓	✗	✓	✓	✓	✓	✗
Benzo (a) anthracene	0.2	✓	✓	✓	✓	✓	✓	✓
Chrysene	0.2	✓	✓	✓	✓	✓	✓	✓
Bis(2-ethylhexyl) phthalate	0.2	✓	✓	✓	✓	✓	✓	✓

Table S9. Target compounds detected in real-world casework soil samples by the laboratory GC-MS method, including concentrations based on dry weight, and by the portable GC-MS. Only those compounds detected by either technique are listed.

Sample	Compound	Laboratory result (ppm)	Detected by portable GC-MS
1	Phenanthrene	1.6	Yes
	Fluoranthene	2.9	Yes
	Pyrene	3	Yes
	Benzo/Chrysene	1.3	Yes
2	Phenanthrene	0.94	**No**
	Fluoranthene	1.7	No
	Pyrene	1.6	No
	Benzo/Chrysene	0.78	No
3	Phenanthrene	1.2	No
	Fluoranthene	2.2	No
	Pyrene	2	No
	Chrysene	1	No
4	Phenanthrene	1.3	Yes
	Fluoranthene	2.5	Yes
	Pyrene	2.4	Yes
	Benzo/Chrysene	1.2	No
5	Fluorene	1.1	No
	Fluoranthene	1.1	Yes
	Pyrene	1.2	Yes
	Benzo/Chrysene	0.77	No
6	Phenanthrene	1.5	Yes
	Fluoranthene	2.6	Yes
	Pyrene	3.5	Yes
	Benzo/Chrysene	2.1	Yes
7	Fluoranthene	0.84	Yes
	Pyrene	0.91	Yes
	Benzo/Chrysene	0.50	Yes
8	Phenanthrene	0.90	Yes
	Fluoranthene	1.2	Yes
	Pyrene	1.3	Yes
	Benzo/Chrysene	0.69	Yes
9	Phenanthrene	0.77	Yes
	Fluoranthene	1.5	Yes
	Pyrene	1.9	Yes
	Benzo/Chrysene	1.2	YEs
10	Phenanthrene	0.98	Yes
	Fluoranthene	1.9	Yes
	Pyrene	2.3	Yes
	Benzo/Chrysene	1.4	Yes
11	Phenanthrene	1.2	Yes
	Fluoranthene	1.9	Yes
	Pyrene	2.4	Yes
	Benzo/Chrysene	1.4	Yes
12	Phenanthrene	3.1	Yes
	Fluoranthene	3.7	Yes
	Pyrene	4	Yes
	Benzo/Chrysene	2.1	yes
13	Phenanthrene	2.5	Yes
	Anthracene	0.72	Yes
	Fluoranthene	4.2	Yes
	Pyrene	6.9	Yes

	Benzo/Chrysene	5.1	No
14	Phenanthrene	2.9	Yes
	Anthracene	0.81	Yes
	Fluoranthene	4.2	Yes
	Pyrene	4.4	Yes
	Benzo/Chrysene	2.3	Yes
15	Phenanthrene	1.2	Yes
	Fluoranthene	2.4	Yes
	Pyrene	2.9	Yes
	Benzo/Chrysene	1.5	Yes
16	Phenanthrene	1.1	Yes
	Fluoranthene	2	Yes
	Pyrene	2.3	Yes
	Benzo/Chrysene	1.4	Yes
17	Fluoranthene	0.50	Yes
	Pyrene	0.50	Yes
18	Phenanthrene	1.3	Yes
	Fluoranthene	2.6	Yes
	Pyrene	2.9	Yes
	Benzo/Chrysene	1.5	Yes
19	Fluoranthene	0.50	Yes
	Pyrene	0.55	Yes
20	Naphthalene	2.4	Yes
	Acenaphthene	0.46	Yes
	Phenanthrene	2.3	Yes
	Anthracene	0.66	No
	Fluoranthene	2.2	Yes
	Pyrene	2.5	Yes
	Benzo/Chrysene	1.4	Yes
21	Phenanthrene	1.1	Yes
	Anthracene	1.3	Yes
	Fluoranthene	2.9	Yes
	Pyrene	3.1	Yes
	Benzo/Chrysene	1.4	Yes
22	M+p-cresol	21	yes
	Naphthalene	2.6	No
	Bis(2-ethylhexyl) phthalate	24	Yes
23	o-cresol	NA	Yes
	M+p-cresol	0.9	Yes
24	Phenanthrene	4.6	Yes
	Anthracene	1.6	No
	Fluoranthene	9.9	Yes
	Pyrene	11	Yes
	Benzo/chrysene	5.0/4.5	yes
25	Phenanthrene	6.4	Yes
	Anthracene	1.9	Yes
	Fluoranthene	15	Yes
	Pyrene	16	Yes
	Benzo/chrysene	7.3/6.3	Yes
26	Fluorene	0.51	No
	Phenanthrene	3.9	Yes
	Anthracene	1.2	No

	Fluoranthene	6	Yes
	Pyrene	5.6	Yes
	Benzo/chrysene	2.6/2.3	Yes
27	Phenanthrene	3.9	Yes
	Anthracene	1.2	No
	Fluoranthene	7.6	Yes
	Pyrene	7.4	Yes
	Benzo/chrysene	3.2/2.9	Yes
28	Phenanthrene	3.4	Yes
	Anthracene	1.0	No
	Fluoranthene	7.4	Yes
	Pyrene	7.4	Yes
	Benzo/chrysene	3.6/3.2	Yes
29	Acenaphthene	0.78	Yes
	Phenanthrene	3.5	Yes
	Anthracene	1.3	Yes
	Fluoranthene	11	Yes
	Pyrene	9.8	Yes
	Benzo/chrysene	4.8/4.2	Yes
30	Phenanthrene	3.3	Yes
	Anthracene	1	No
	Fluoranthene	6.2	Yes
	Pyrene	5.9	Yes
	Benzo/chrysene	2.7/2.4	Yes
31	Fluorene	0.54	Yes
	Phenanthrene	4.6	Yes
	Anthracene	1.5	No
	Fluoranthene	8.4	Yes
	Pyrene	7.8	Yes
	Benzo/chrysene	3.7/3.4	Yes
32	Fluoranthene	1.1	Yes
	Pyrene	1.4	Yes
	Benzo/chrysene	0.72/0.67	No
33	Phenanthrene	0.84	Yes
	Fluoranthene	2.7	Yes
	Pyrene	3.4	Yes
	Benzo/chrysene	1.6/1.4	Yes
34	Fluoranthene	0.94	Yes
	Pyrene	1.5	Yes
	Benzo/chrysene	0.58/0.55	No
35	Fluoranthene	1	Yes
	Pyrene	1.3	Yes
	Benzo/chrysene	0.67/0.58	No
36	Fluoranthene	1.8	Yes
	Pyrene	1.8	Yes
	Benzo/chrysene	0.75/0.75	No
37	Fluoranthene	1.5	Yes
	Pyrene	1.6	Yes
	Benzo/chrysene	0.76/0.71	No
38	Fluoranthene	1	Yes
	Pyrene	1.2	Yes
	Benzo/chrysene	0.61/0.62	No
39	Phenanthrene	1.3	Yes

	Fluoranthene	2.7	Yes
	Pyrene	3	Yes
	Benzo/chrysene	1.5/1.3	Yes
40	Phenanthrene	1.4	Yes
	Fluoranthene	4.2	Yes
	Pyrene	4.4	Yes
	Benzo/chrysene	2.5/2.1	Yes
41	Phenanthrene	1.4	Yes
	Fluoranthene	3.9	Yes
	Pyrene	4	Yes
	Benzo/chrysene	2.1/1.7	No
42	Phenanthrene	2.4	Yes
	Anthracene	0.75	Yes
	Fluoranthene	5	Yes
	Pyrene	4.5	Yes
	Benzo/chrysene	2.3/2.0	Yes
43	Phenanthrene	0.90	Yes
	Fluoranthene	2.7	Yes
	Pyrene	2.6	Yes
	Benzo/chrysene	1.5/1.3	Yes
44	Phenanthrene	2.3	No
	Anthracene	0.61	No
	Fluoranthene	4.9	Yes
	Pyrene	4.1	Yes
	Benzo/chrysene	2.3/1.9	No
45	Phenanthrene	2	No
	Anthracene	0.57	No
	Fluoranthene	4.4	Yes
	Pyrene	4	Yes
	Benzo/chrysene	2.1/1.8	No
46	Phenanthrene	1.1	Yes
	Fluoranthene	2.9	Yes
	Pyrene	2.6	Yes
	Benzo/chrysene	1.6/1.3	Yes
47	Phenanthrene	1.9	Yes
	Anthracene	0.56	Yes
	Fluoranthene	3.8	Yes
	Pyrene	3.3	Yes
	Benzo/chrysene	1.8/1.5	Yes
48	Phenanthrene	0.75	Yes
	Fluoranthene	1.8	Yes
	Pyrene	1.9	Yes
	Benzo/chrysene	1.0/0.86	No
49	Phenanthrene	0.86	Yes
	Fluoranthene	1.9	Yes
	Benzo/chrysene	1.0/0.89	Yes
50	Phenanthrene	0.58	No
	Fluoranthene	1.5	Yes
	Benzo/chrysene	0.84/0.71	Yes

Table S10. Target compounds detected in real-world casework water samples via the laboratory GC-MS method, including concentrations, and by the portable GC-MS. Only those compounds detected by either technique are listed.

Sample	Compound	Laboratory result (ppm)	Detected by portable GC-MS
1	3+4-Methylphenol (m+p-cresol)	21	Yes
	Naphthalene	2.6	No
	Bis(2-ethylhexyl) phthalate	24	Yes
2	3+4-Methylphenol (m+p-cresol)	0.9	Yes
3	Naphthalene	0.22	Yes
4	Naphthalene	80	Yes
5	2-methylphenol	0.12	Yes
	3+4-methylphenol	0.22	Yes
	2,4-dimethylphenol	0.06	Yes
6	2-methylphenol	N/A	Yes
	3+4-methylphenol	N/A	Yes
	2,4-dimethylphenol	N/A	Yes
